# Unveiling Halogen-Bonding Interactions between a Pyridine-Functionalized Fluoroborate Dye and Perfluorohaloarenes with Fluorescence Spectroscopy

**DOI:** 10.1021/acs.joc.2c01660

**Published:** 2022-11-01

**Authors:** Alex Iglesias-Reguant, Judyta Zielak-Milewska, Tomasz Misiaszek, Robert Zaleśny, Josep M. Luis, Borys Ośmiałowski

**Affiliations:** † Faculty of Chemistry, Nicolaus Copernicus University, Gagarina 7, Toruń PL-87100, Poland; ‡ Faculty of Chemistry, Wrocław University of Science and Technology, Wyb. Wyspiańskiego 27, Wrocław PL-50370, Poland; § Institute of Computational Chemistry and Catalysis and Department of Chemistry, University of Girona, Campus de Montilivi, Girona, Catalonia 17071, Spain

## Abstract

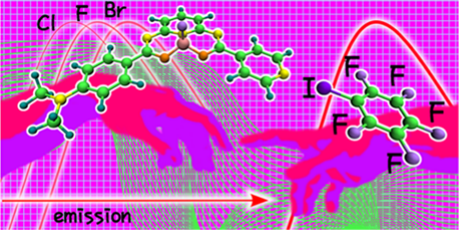

We have studied the halogen-bonding interactions of a pyridine-functionalized fluoroborate dye with perfluorohaloarenes (C_6_F_6_, C_6_F_5_Cl, C_6_F_5_Br, and C_6_F_5_I) in the two-component-only liquid phase using fluorescence spectroscopy. Based on the results of spectroscopic measurements and electronic-structure calculations, we have confirmed the stability only for the complex between C_6_F_5_I and the emissive dye, and it has been demonstrated that halogen-bonding interactions are accompanied by significant Stokes shifts for the ππ* band. We also provide experimental evidence that for this complex, the emission is quenched due to a simultaneous decrease of radiative and increase of nonradiative decay rate constants upon halogen-bonding interactions.

## Introduction

Studies of halogen bonding (XB) are in the limelight as many areas benefit from its properties, including crystal engineering^1−4^ and live sciences.^5−7^ More specifically, XB-based co-crystallization,^8−10^ construction of liquid crystals-based^11^ phosphorescent,^12^ anion transport,^13,14^ and sensing^15^ materials are only a few examples of many applications of those non-covalent weak intermolecular forces. A very recent study shows how important the halogen bonding is for the aggregation of photoactive compounds with attached luminophores.^16^ Several reviews focused on XB highlight the dependence of this interaction on electron acceptors in the molecule carrying the halogen bond donor.^17−19^ Although XB has great potential in crystal engineering in the design of new materials,^20^ understanding its properties in solution delivers a complementary information on their nature. This was the rationale behind numerous studies in solutions, often composed of more than two constituents, which aimed at understanding the thermodynamics of the XB formation or changes in vibrational spectra induced by the XB.^6,21−25^ The fundamental aspects of XB in simple systems are nowadays well understood and the challenge lies in obtaining the full benefit of this knowledge in rational design of materials with tailored properties. In particular, modifying the optical responses of π-conjugated dyes, including their emission, by specific interactions such as XB is a particularly attractive design route. A recent study demonstrated that, for heavy-atom carrying diiodo-BODIPY interacting with XB acceptors^26^ or in bromobenzaldehyde in the solid state,^27^ halogen bonding facilitates the formation of the triplet state after the photon absorption by increasing the intersystem crossing rate constant. On the other hand, in the case of anion sensing compounds, the formation of the XB complex increases the fluorescence of the probe due to rigidification of its structure^15,28^ or causes a serious limitation of random movements within, for example, the interlocked catenanes.^29^

In order to fully benefit from properties of halogen-bonding interactions involving organic dyes, it is mandatory to understand the subtle changes in the electronic structure of organic compounds upon the formation of a halogen bond. Moreover, considering that the absolute value of interaction energy in complexes stabilized by XB follows the F < Cl < Br < I ordering due to different polarizabilities of halogen (fluorine is rarely involved in such interactions^30,31^), hence, studying the whole series of halogen atoms delivers thorough characteristics of XB properties. The present study contributes to these efforts and aims at studying the changes in electronic absorption and emission spectra upon interactions of an emissive pyridine-functionalized dye with perfluorohaloarenes (C_6_F_6_, C_6_F_5_Cl, C_6_F_5_Br, and C_6_F_5_I) in the two-component-only liquid phase. In fact, previously it has been shown that electronic spectroscopy can be useful in the study of halogen bond in molecular complexes.^32^

**Figure 1:**
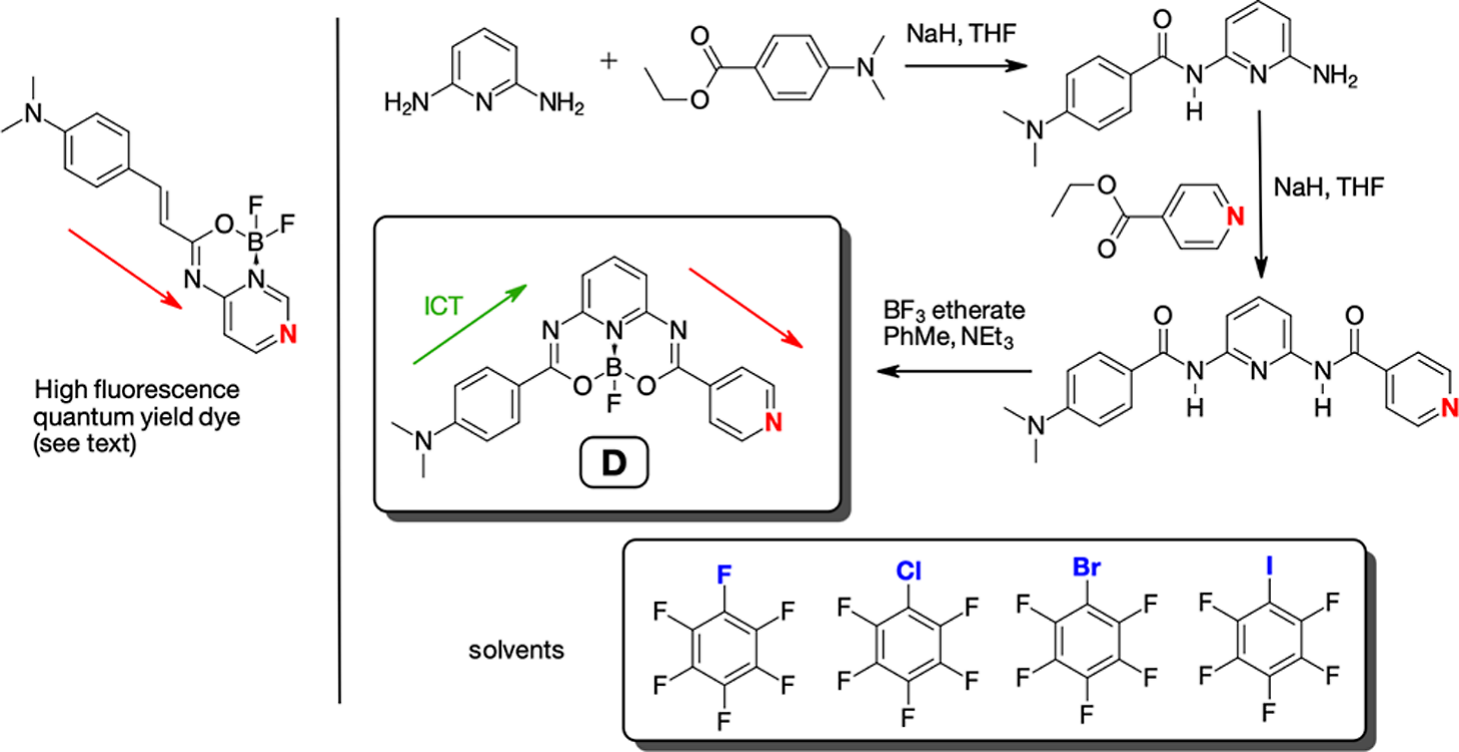
Synthesis of studied emissive dye (**D**) and the palette of solvents. The green arrow indicates the direction of the ICT, and the red arrows indicate the direction of the donor-to-acceptor conjugation paths.

It is known that a very efficient BF_2_-/BF-carrying fluorophores (similar to classical BODIPYs) hold a privileged spot due to high fluorescence quantum yields (FQYs) deriving from a rigid core in their structure. Similar rigidity, crucial for enhancing the radiative dissipation of excitation energy, is the characteristics of several close-relative families of dyes: boranils,^33,34^ ketonates,^35,36^ or ketoiminates.^36,37^ Given the excellent photophysical properties of these dyes, we designed the new compound (denoted as **D**) shown in Figure 1. Because the topology of the dye is crucial, during the design of **D**, we relied on the experience gained in some of our previous works. The structure of **D** was proposed to obtain a molecule prone to interactions through XB and exhibiting intramolecular charge transfer (ICT) in the lowest-lying electronic excited state. Note that, by design, the halogen bond acceptor is not the primary electron-donating moiety involved in the ICT. Because the highest fluorescence quantum yield in previously described diazines^38^ was obtained for the isomer carrying a nitrogen atom in the para position in relation to the donor-to-acceptor conjugation path (red arrow in Figure 1), in the current study, pyridin-4-yl was used as the building block. Taken together, the structural and photophysical characteristics of **D** should allow for the achievement of the specific goals of this study, including the following: (a) confirming the formation of a halogen bond and (b) analyzing its influence on the photophysical properties of the dye, with special emphasis on the radiative energy dissipation.

The palette of solvents used is also shown in Figure 1. The synthesis of **D** was carried out in a similar way to dyes presented in a previous publication by some of the present authors.^39^ The procedure is described in the Methods and Protocols, but we want to emphasize that unlike in other studies aiming at characterizing XB interactions in solutions based on absorption and emission, we used a non-competitive environment without any additional solvent. Furthermore, all spectra reported here were recorded in solvents freshly distilled under a nitrogen atmosphere.

## Results and Discussion

**Figure 2:**
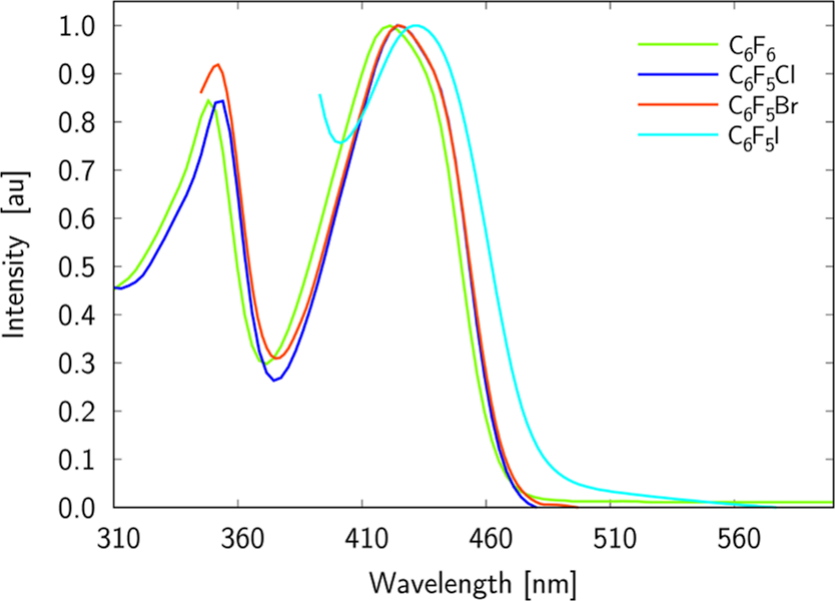
Normalized absorption spectra of **D** in C_6_F_6_, C_6_F_5_Cl, C_6_F_5_Br, and C_6_F_5_I solvents.

**Figure 3:**
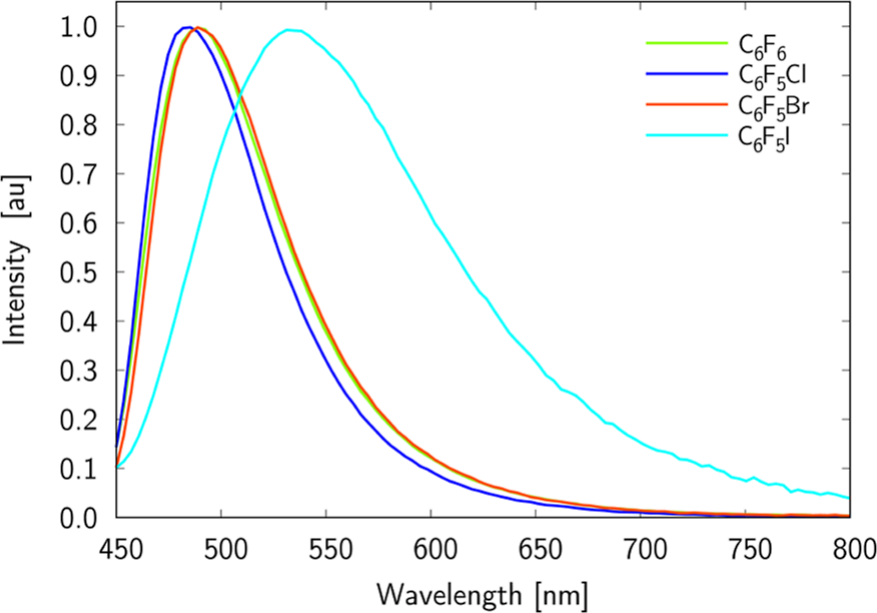
Normalized fluorescence spectra of **D** in C_6_F_6_, C_6_F_5_Cl, C_6_F_5_Br, and C_6_F_5_I solvents.

Figures 2 and 3 show the electronic absorption and emission spectra of **D** in C_6_F_6_, C_6_F_5_Cl, C_6_F_5_Br, and C_6_F_5_I solvents, while the summary of the photophysical properties of such spectra is collected in Table 1.

**Table 1:** Photophysical Properties of **D** in Various Solvents Measured at r.t. and Values Marked in Bold Were Calculated at the TD-DFT Level^a^

solvent	λ_abs_ [nm]	ϵ [M^–1^ cm^–1^]	λ_em_ [nm]	Stokes [cm^–1^]	FQY [%]	τ [ns]	*k*_r_ [10^9^ s^–1^]	*k*_nr_ [10^9^ s^–1^]	χ^2^
C_6_F_6_	420	20,290	490	3400	35.1	2.91	0.121	0.223	1.101
C_6_F_5_Cl	425	20,270	484	2870	39.8	2.97	0.134	0.203	1.042
	**382**		**437**	**3293**					
C_6_F_5_Br	425	20,330	493	3245	34.1	2.59	0.130	0.254	1.075
	**385**		**454**	**3938**					
C_6_F_5_I	430	20,620	539	4702	1.4	0.51	0.027	1.933	1.364
	**388**		**473**	**4623**					

a The τ for C_6_F_5_I is the weighted average of two lifetimes, τ = 0.48 (*α* = 0.94) and τ = 1.01 ns (α = 0.06).

**Figure 4:**
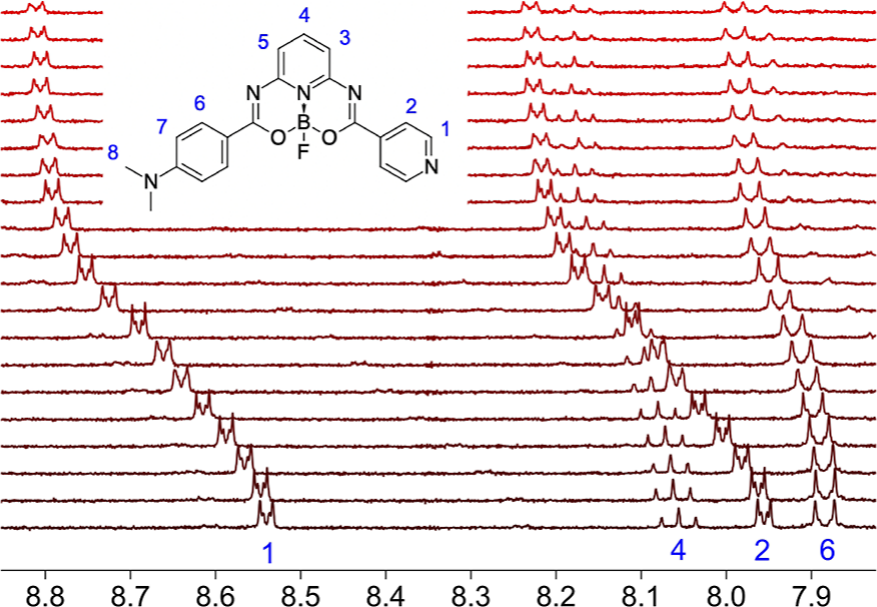
NMR stacked spectra for titration of **D** by C_6_F_5_I in C_6_F_6_ solution.

The cut of the absorption spectra at ca. 390 and 345 nm is due to the intense absorption of C_6_F_5_I and C_6_F_5_Br, respectively. As it is expected for ICT-exhibiting molecules, the absorption spectra measured for the four used solvents have a broad absorption band without any vibrational fine structure. A clear noticeable red-shift of absorption band maximum with respect to that for C_6_F_6_ is noticed solely for C_6_F_5_I. This trend is even more pronounced in the case of emission spectra, that is one finds a much broader and red-shifted band for **D** in C_6_F_5_I. Note that shifts of emission bands for other solutions are insignificant with respect to C_6_F_6_. Although C_6_F_5_Br and C_6_F_5_I are the two most similar solvents of the set, Figures S8–S10 in the Supporting Information file demonstrate that the changes in emission intensity between C_6_F_5_Cl/C_6_F_5_Br and C_6_F_5_I solutions at various temperatures are significant. More specifically, the spectra recorded in C_6_F_5_I are far more sensitive to the temperature changes than the C_6_F_5_Br ones. This could be rationalized by considering that C_6_F_5_I is the only solvent that has a stable XB interaction with the heterocyclic nitrogen atom present in the pyridin-4-yl moiety. The N···I interactions are further supported by the FTIR spectra shown in Figure S11 in the Supporting Information file and the ^1^H NMR titrations of **D** in C_6_F_6_ by C_6_F_5_I (Figures S12–S14, see Supporting Information file), which deliver the association constant of approximately 750 M^–1^. Moreover, the XB interaction between C_6_F_5_I and **D** was confirmed by the comparison of the CIS (*complexation-induced shift*, in [ppm], defined as the difference between chemical shift of the respective proton in the free molecule and in the interacting molecule at the saturation conditions) for every proton in **D** (Figure S15). The description of the experimental procedure is available in the Methods and Protocols section, while the exemplified, stacked NMR spectrum, with labeled protons, is shown in Figures 4 and S12.

Taken together, these results confirm that **D** in its ground electronic state interacts specifically with C_6_F_5_I. It is worth highlighting that the emission for **D** at a low temperature is less intense than at room or elevated temperatures, a feature that is in contrast to the usual temperature effect on fluorescence. We suggest that this temperature dependence is caused by the increase of the strength of XB between **D** and C_6_F_5_I when the temperature decreases (observed even in rigid solid^40^). The fluorescence lifetime measurements at room temperature (Table 1) show that the drop of fluorescence quantum yield upon C_6_F_5_I/**D** complex formation is caused by a 5-fold decrease in *k*_r_ and 10-fold increase in *k*_nr_ values.

In order to further support the experimental pieces of evidence of influence of halogen bonding on the electronic spectra of **D**, we performed a series of electronic-structure calculations using (time-dependent) density functional theory [(TD) DFT] with the MN15 functional and wave function-based SCS-MP2 method. The computational details are presented in the Methods and Protocols section. The geometry optimizations in the ground electronic state indicate that C_6_F_6_ does not form a XB with the nitrogen atom of the pyridine in **D**. The calculated ground-state interaction energies for the remaining complexes are shown in Table S2, and they follow the usual trend for halogen-bonded complexes, that is, the value of interaction energy for C_6_F_5_I/**D** corresponds to the most stable complex. Comparison of the interatomic distances within the N···X bridge through the different compounds in the ground and excited equilibrium geometries leads to interesting conclusions. In Table 2, the distance between the halogen atom and the nitrogen acting as a halogen bond acceptor is shown for the ground state (S_0_) and the excited state (S_1_) geometry.

**Table 2:** Bond Lengths (in Å) and Angles (in deg) for the Electronic Ground (S_0_) and Excited State (S_1_)^a^

	N···X bond length (R, [Å])			N···X–C bond angle (α, [deg])	
complex	R(S_0_)	R(S_1_)	R(S_1_)-R(S_0_)	α(S_0_)	α(S_1_)
C_6_F_5_Br/D	2.8290	2.7164	–0.1126	174.22	179.94
C_6_F_5_I/D	2.8057	2.6827	–0.1230	179.74	179.66

a Geometry optimizations were performed at the MN15/aug-cc-pVDZ(PP) level of theory.

**Figure 5:**
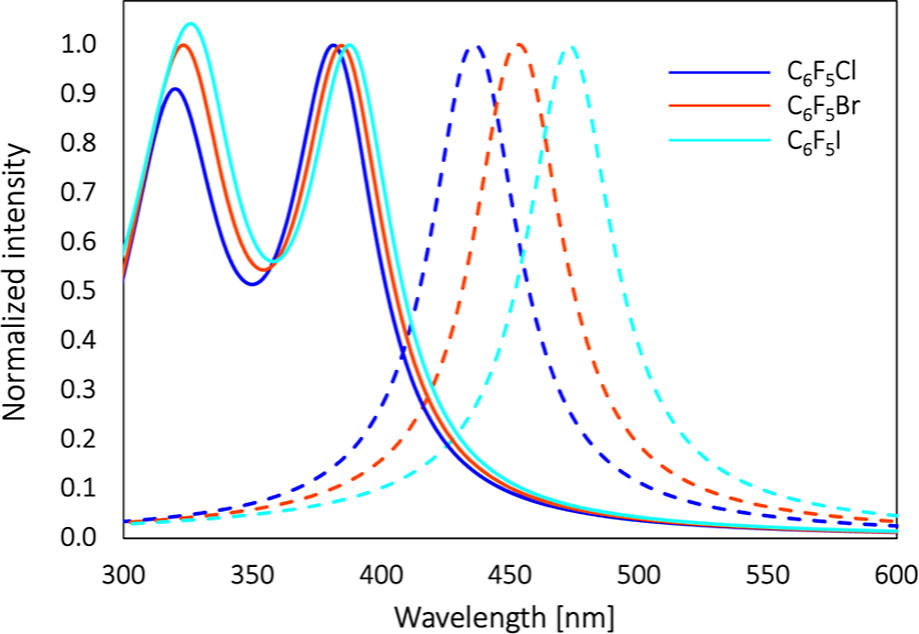
Normalized simulated absorption (solid line) and emission (dashed line) spectra for C_6_F_5_Cl/**D**, C_6_F_5_Br/**D**, and C_6_F_5_I/**D** complexes in the gas phase at the MN15/aug-cc-pVDZ level of theory.

For both states, the N···X length decreases while increasing the polarizability of the halogen involved in the interactions, following the interaction energy values. Moreover, Table 2 shows that on passing from the ground electronic state to the excited state, a shortening of N···X is observed for all complexes, with largest (smallest) shortening found for C_6_F_5_I/**D** (C_6_F_5_Cl/**D**). Table 2 also contains the angle of the halogen bond for the different compounds, and it can be observed that the stronger the interaction, the closer is the angle to 180°. The trends in DFT-based absorption spectra of all isolated complexes are in line with the experimental data. Notably, those calculations show that all compounds exhibit absorption features in the range of 380–390 nm (see Table 1). Interestingly, these results show that complex C_6_F_5_I/**D** presents a small but clear red shift in the maximum of absorption (6 nm) with respect to C_6_F_5_Cl/**D**. However, the simulated spectra of all complexes (Figure 5) are very similar, a feature that supports the general rule of the larger sensibility of emission spectra over absorption to the changes in the electronic structure of **D**.

**Figure 6:**
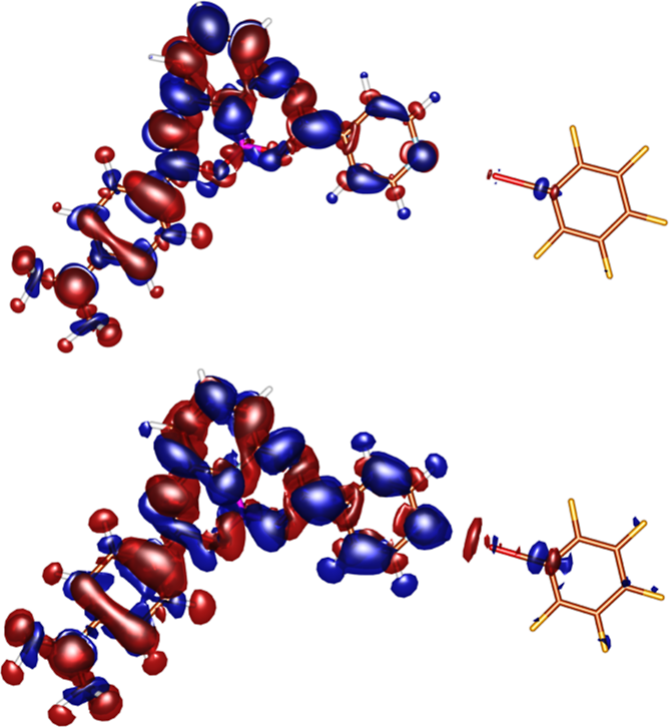
Electron density difference between the excited and ground sates of the absorption (top) and emission (bottom) process for the **D**/C_6_F_5_I complex.

We have also performed TD-DFT calculations to take into account the effect of the C_6_F_6_ and C_6_F_5_I solvent polarity on emission spectra using the SMD implicit solvation model. The TD-DFT calculations predict a shift between the emission of **D** in both solvents of only 7 nm, indicating that the experimental shift cannot be explained considering only the effect of the solvent polarity. In order to link these observations with the electronic structure of the complexes, we determined the difference in electronic density between the excited and ground state at the ground- and excited-state geometry (Figure 6). The results show that electronic density difference, pointing toward vertical absorption, is not large, that is, the effect of the halogen bond on the absorption process is small. On the contrary, there is a significant density change over the N···I bond during the vertical emission process, explaining the sensitivity of the emission wavelength due to the formation of the halogen bond. We note a satisfactory qualitative agreement between the simulated emission spectra of all complexes and the experimental values. The emission wavelength follows the trend of halogen bonding strengths (Cl < Br < I), with the latter being the one that presents a larger red shift with respect to C_6_F_6_/**D**. Another feature observed is the decrease of the intensity in the emission bands while following the halogen bond strength trend. This fact is in accordance with the measured fluorescence quantum yield. Although all these features agree between experiments and simulations, a distinct difference in the C_6_F_5_Br/**D** emission spectra is observed. From experiments, the shift of the maximum of emission in C_6_F_5_Br is not large, while in DFT calculations, it is more pronounced, agreeing once more with the trend of the halogen bonding strength order. To shed light on this observation, we computed the Gibbs energy (Δ*G*) for the formation of C_6_F_5_Cl/**D**, C_6_F_5_Br/**D**, and C_6_F_5_I/**D** complexes (Table 3), including the correction term to simulate the effect of the experimental concentration of the reactants.

**Table 3:** Formation Gibbs Energy (Δ*G*, in kcal/mol) and Formation Energy (Δ*E*, in kcal/mol) Calculated Using the MN15 Functional and the Aug-cc-pVDZ(PP) Basis Set

complex	Δ*G*	Δ*E*
C_6_F_5_Cl/D	3.181	–2.478
C_6_F_5_Br/D	0.493	–5.017
C_6_F_5_I/D	–1.717	–8.029

The only complex that presents an exergonic complex formation due to the halogen bonding is C_6_F_5_I/**D**. Therefore, the DFT calculations suggest that only the formation of the C_6_F_5_I/**D** complex is favorable. On the contrary, in the experimental conditions, DFT calculations indicate that C_6_F_5_Cl/**D** and C_6_F_5_Br/**D** complexes are not favorable. These data support the experimental results showing the largest shifts in absorption and emission spectra for the C_6_F_5_I/**D** complex.

## Conclusions

In conclusion, we have studied the halogen-bonding interactions between an emissive pyridine-functionalized fluoroborate dye and perfluorohaloarenes (C_6_F_6_, C_6_F_5_Cl, C_6_F_5_Br, and C_6_F_5_I) in the two-component-only liquid phase. Based on the spectroscopic measurements, supported by electronic-structure calculations, we have confirmed the stability of the C_6_F_5_I/**D** complex, and it has been demonstrated that only halogen-bonding between **D** and C_6_F_5_I is accompanied by significant Stokes shifts for the ππ* band. This effect is not observed for other studied complexes. We also provided experimental evidence that in the case of the C_6_F_5_I/**D** complex, the emission is quenched due to the decrease of *k*_r_ and increase of *k*_nr_ upon halogen-bonding interactions at a specific site of the dye. The fluorescence quantum yield is even more influenced by temperature. Even though electronic spectroscopy was more scarcely used in studies of halogen bonding in solution in comparison to other techniques, the results presented herein demonstrate that this technique can provide valuable information regarding the influence of XB on photophysical properties of dyes in the two-component-only liquid phase.

## Methods and Protocols

### Synthesis

The synthetic path shown in the article starts with the synthesis of *N*-(6-aminopyridin-2-yl)-4-(dimethylamino)benzamide (mono-amide) by the reaction between 2,6-diaminopyridine and ethyl 4-(dimethylamino)benzoate in THF solution with the use of two equivalents of sodium hydride (60% suspension in oil) as in one of our previous reports.^41^ The obtained mono-amide was used in the next step, i.d. during the formation of another amide moiety as follows: to a THF solution of *N*-(6-aminopyridin-2-yl)-4-(dimethylamino)benzamide (1.0 g, 3.9 mmol) in three-necked flask, two equivalents of NaH (ca. 0.32 g, 60% suspension in oil) was added under a dry nitrogen flow. After magnetic stirring (2 h) at boiling point, one equivalent of ester (0.59 g, 3.9 mmol, solution in 5 mL of dry THF) was added using a syringe through the septum. The mixture was heated at boiling point overnight. After cooling to r.t., the reaction was quenched with saturated, water NH_4_Cl solution (10 mL). The resulting mixture was evaporated to remove THF, solid material that precipitated from water separated, and washed gently with cold water to remove inorganic salts. The residual was recrystallized from alcohol to give 0.49 g (34.8%) of the final bis-amide. The resulted bis-amide was treated with BF_3_ etherate (5 equiv, ca. 0.85 mL)^41^ in boiling toluene (20 mL) and 5 equiv of DIEA (ca. 1.1 mL) was added. After heating the reaction mixture for 5 h, the reaction was cooled and quenched with saturated, water Na_2_CO_3_ solution (10 mL). The mixture was extracted with DCM, the organic layer was dried using Na_2_SO_4_, evaporated, and purified using column chromatography (eluent DCM), giving 0.16 g (30.3%) of pure **D**.

### Measurements

All of the NMR spectra were recorded using a 400 MHz Bruker spectrometer at room temperature (spectra available in the Supporting Information). Structural assignments were made with additional information from gCOSY, gHSQC, and gHMBC experiments. HRMS spectrum was recorded using sector mass spectrometer AutoSpec Premier (Waters) equipped with an electron impact (EI) ion source and the EBE double focusing geometry mass analyzer. Absorption measurements were carried out at r.t. with the use of a Shimadzu UV-1900 spectrophotometer using cuvettes with 1 cm light path. For the determination of molar attenuation coefficient, solutions of known concentration with absorbance between 0.2 and 0.9 were prepared and, based on the linear correlation, the attenuation coefficient was found (correlation coefficient for linear fit higher or equal to *R*^2^ = 0.99). The emission spectra were recorded with a FS5 (Edinburgh Instruments) spectrofluorimeter equipped with thermostated holder (thermoelectric cooling by Peltier device) joined with cooling. The excitation was realized at maximum of absorption for solutions characterized by the maximum absorbance of ca. 0.1. The fluorescence quantum yields were measured with the use of the integrating sphere (add-on for FS5). The fluorescence lifetimes were recorded with the same spectrofluorimeter using the TCSPC technique and high repetition (ps) pulsed light source (excitation at 450 nm, emission at λ_em_). All solvents were distilled under a nitrogen atmosphere directly before their use in measurements. To avoid the presence of oxygen and humidity in the measurements chamber, the slow flow of the dry nitrogen was supplied. That was especially important during low temperature measurements. The stabilization time for spectra recorded at variable temperatures was set to 3 min after reaching the needed temperature of the holder.

### Characterization Data

*N*-{6-[*p*-(Dimethylamino)benzylamino]-2-pyridyl}isonicotinamide (a bis-amide substrate for the synthesis of **D**, Figure 1) ^1^H NMR (400 MHz, DMSO): δ 10.88 (s, 1H), 10.05 (s, 1H), 8.79 (d, *J* = 6.1 Hz, 2H), 7.95–7.88 (m, 6H), 7.88–7.80 (m, 1H), 6.75 (d, *J* = 9.0 Hz, 2H), 3.01 (s, 6H). ^13^C{^1^H} NMR (DMSO, 101 MHz): δ 165.7, 164.9, 153.1, 151.5, 150.8, 150.4, 141.6, 140.4, 129.9, 122.2, 120.3, 111.9, 111.3, 111.2. The signal of the methyl group overlapped with the solvent residual signal. Still, the methyl group is clearly visible in the ^13^C NMR spectrum of the final product (**D**) recorded in CDCl_3_ (below). ^15^N NMR (40.5 MHz, DMSO, HMBC): δ 55.4, 133.6, 139.0, 265.7, 326.0. mp 290 °C (decomposition). Gray-yellowish powder.

*N*,*N*-Dimethyl{*p*-[3a-fluoro-5-(4-pyridyl)-3,4-dioxa-1,6,9b-triaza-3a-bora-2-phenalenyl]phenyl} amine (**D**): ^1^H NMR (400 MHz, CDCl_3_): δ 8.80 (d, *J* = 6.1, 2H), 8.23 (d, *J* = 9.1, 2H), 8.16 (d, *J* = 6.1, 2H), 7.98 (t, *J* = 8.1, 1H), 7.17 (dd, *J* = 8.3, 1.0, 1H), 7.11 (dd, *J* = 8.0, 1.0, 1H), 6.71 (d, *J* = 9.2, 2H), 3.10 (s, 6H). ^11^B NMR (128 MHz, CDCl_3_): δ 1.22 (d, *J* = 33.5 Hz). ^13^C {^1^H} NMR (101 MHz, CDCl_3_): δ 166.4, 162.8, 154.0, 151.5, 150.4, 148.9, 144.8, 140.6, 131.9, 122.6, 118.7, 118.0, 115.6, 111.0, 40.3. ^15^N NMR (40.5 MHz, CDCl_3_, HMBC): δ 59.3, 178.9, 210.6, 216.5, 320.5. mp 234–236 °C. Dark red powder. HRMS (EI) *m*/*z*: [M^+^] calcd for C_20_H_17_BFN_5_O_2_, 389.1459; found, 389.1454.

### Computational Details

Ground- and excited-state geometry optimizations were performed using the MN15 functional and aug-cc-pVDZ basis set (for bromine and iodine the aug-cc-pVDZ-PP basis set and the corresponding pseudopotential was used), followed by Hessian evaluation to confirm that the optimized geometries correspond to energy minima. The optimized geometries were used in the electronic-structure calculations using time-dependent density functional theory to determine absorption and emission spectra and electronic density difference plots. Likewise, the MN15/aug-cc-pVDZ(PP) level of theory was used to that end. The formation energy was calculated as the difference between the energy of the complex and the energy of the monomers in the monomer-centered basis set at the MN15/aug-cc-pVDZ(PP) level of theory. The formation Gibbs energy was calculated at the MN15/aug-cc-pVDZ(PP) level of theory, taking into account the correction when moving from a standard gas state that uses a pressure of 1 atm to a standard solvent state that uses a concentration of X M (where X is the molarity concentration of reagents and products in agreement with experimental data).^42^ The intermolecular interaction energy and the preparation energy were computed using counterpoise procedure at the MN15/aug-cc-pVDZ(PP) level of theory. Interaction energies were also determined at the SCS-MP2/aug-cc-pVDZ(PP) level of theory. All (TD)DFT calculations were performed using Gaussian 16 program,^43^ while SCS-MP2 results were obtained using MOLPRO 2012 software.^44^

## Supporting Information Available

NMR spectra, IR spectra, results of additional spectroscopic measurements at variable temperatures, titration procedure, and additional results of electronic-structure calculations (PDF)
