# Supplementary material for: Unveiling Halogen-Bonding Interactions between a Pyridine-Functionalized Fluoroborate Dye and Perfluorohaloarenes with Fluorescence Spectroscopy
Source: J Org Chem. 2022 Nov 1;87(22):15159–65. doi: 10.1021/acs.joc.2c01660 (PMC9680022; doi:10.1021/acs.joc.2c01660)
Supplement: Supplementary file 1 — jo2c01660_si_001.pdf [file jo2c01660_si_001.pdf]

Supporting Information: Unveiling Halogen-Bonding Interactions Between a Pyridine-Functionalized Fluoroborate Dye and Perfluorohaloarenes with Fluorescence Spectroscopy

Alex Iglesias-Reguant,[†] Judyta Zielak,[†] Tomasz Misiaszek,[‡] Robert Zaleśny,[*,‡] Josep M. Luis,[*,¶] and Borys Ośmiałowski[*,†]

†*Faculty of Chemistry, Nicolaus Copernicus University, Gagarina 7, PL-87100 Toruń, Poland*

‡*Faculty of Chemistry, Wrocław University of Science and Technology, Wyb. Wyspiańskiego 27, PL–50370 Wrocław, Poland*

¶*Institute of Computational Chemistry and Catalysis and Department of Chemistry, University of Girona, Campus de Montilivi, 17071, Girona, Catalonia, Spain*

E-mail: robert.zalesny@pwr.edu.pl; josepm.luis@udg.edu; borys.osmialowski@umk.pl

Contents

1 NMR spectra **S-3**

2 Spectroscopic measurements **S-10**

3 Electronic-structure calculations **S-17**

References **S-32**

1 NMR spectra

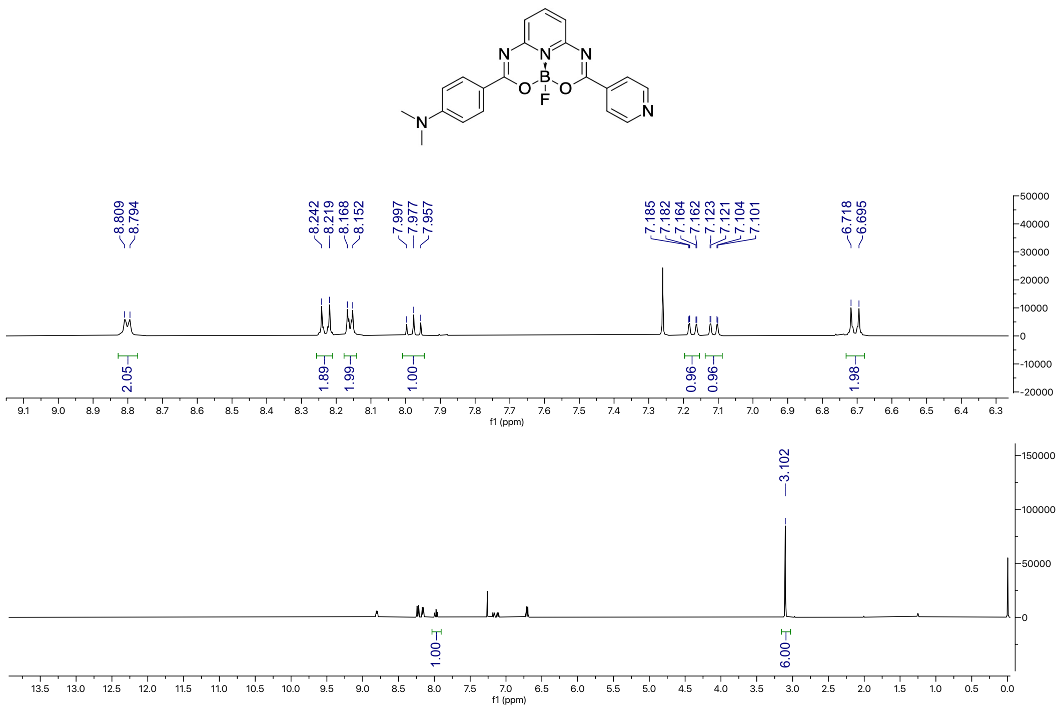

Figure S1: The ^{1}H NMR spectrum of **D** (400 MHz, $CDCl_3$)

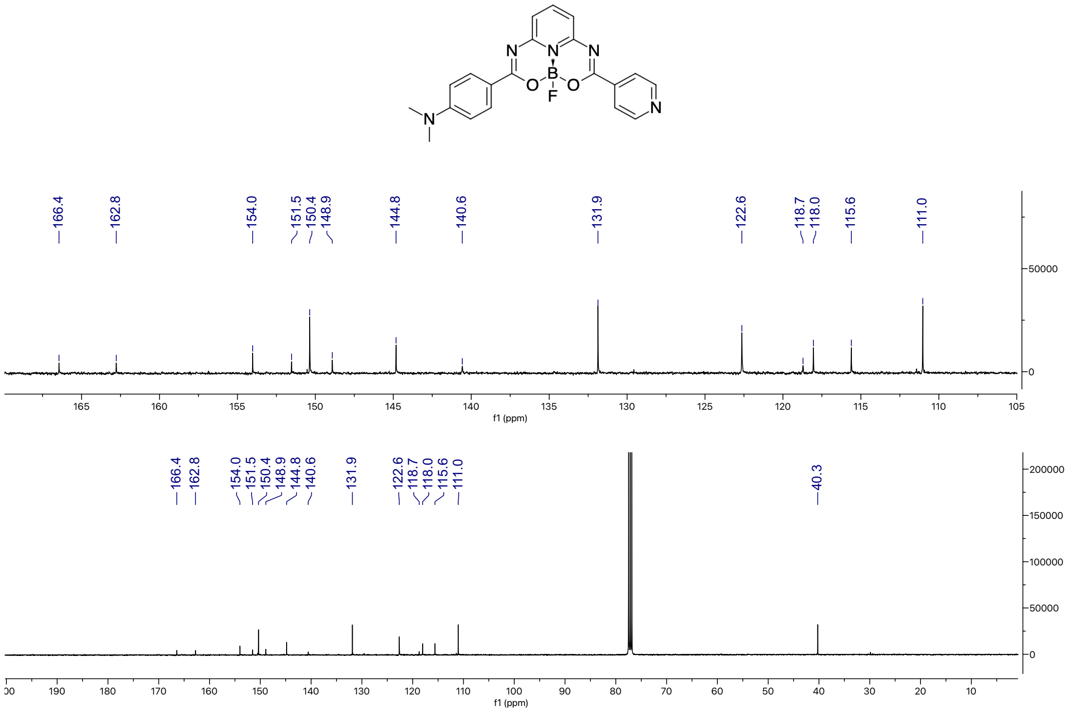

Figure S2: The $^{13}C\{^{1}H\}$ NMR spectrum of **D** (101 MHz, $CDCl_3$)

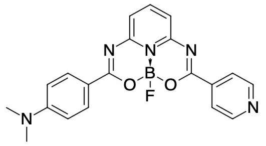

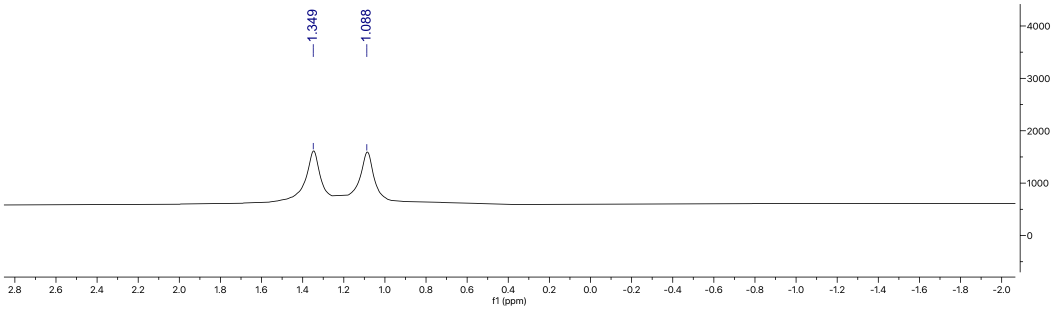

Figure S3: The ^{11}B NMR spectrum of **D** (128 MHz, $CDCl_3$)

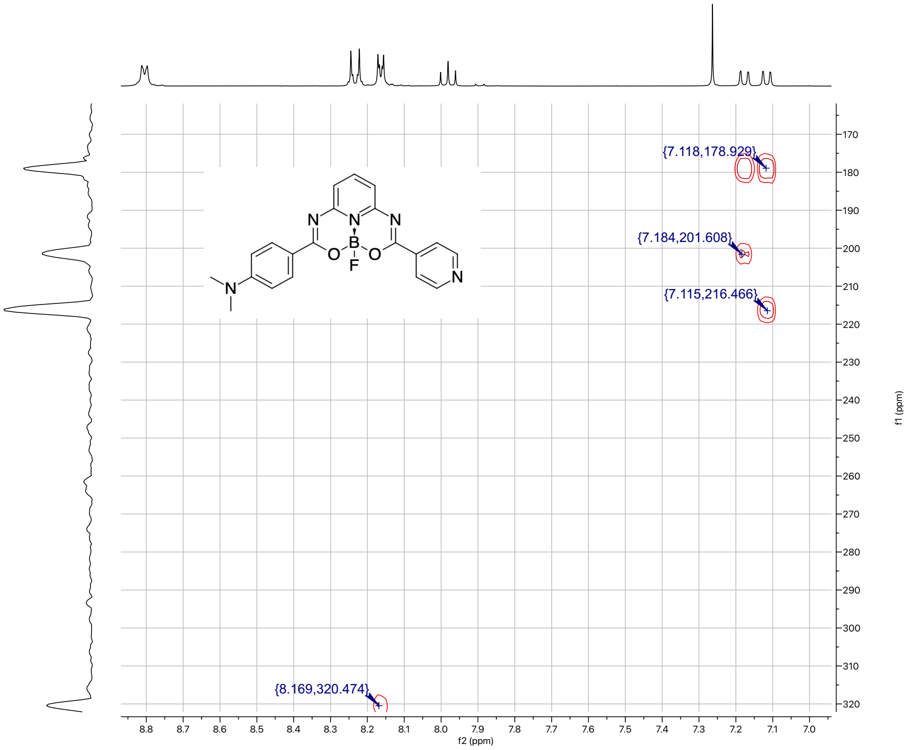

Figure S4: The $^{1}H/^{15}N$ HMBC NMR spectrum of **D** (40.5 MHz, $CDCl_3$)

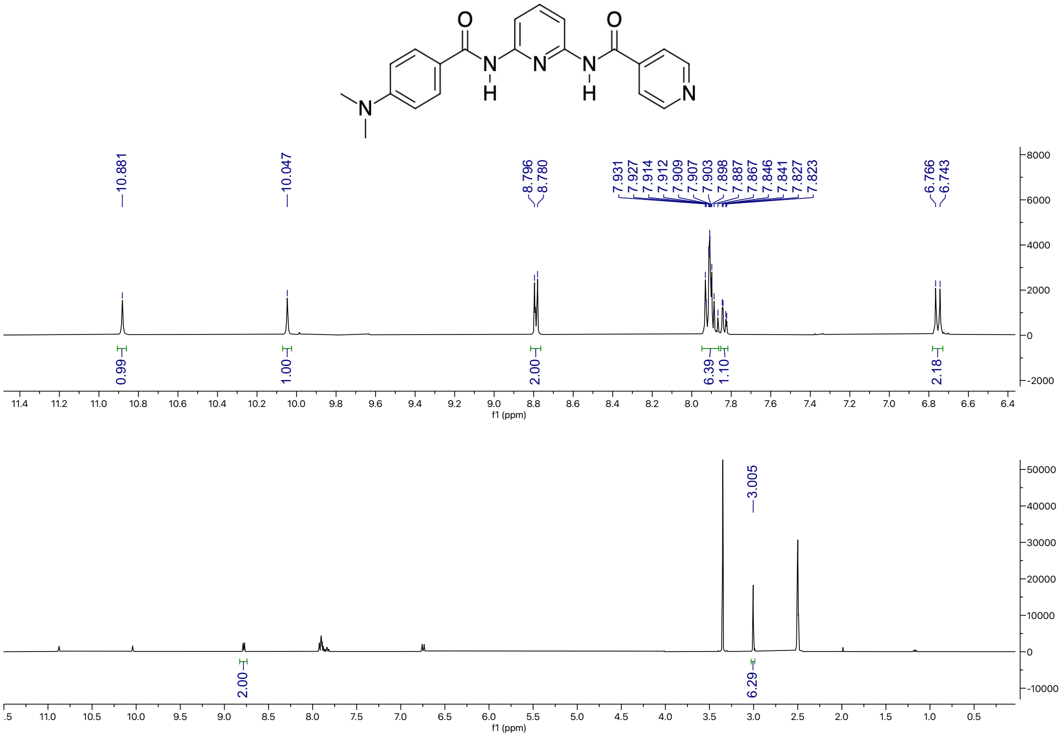

Figure S5: The ^{1}H NMR spectrum of bis-amide (400 MHz, DMSO-d_6)

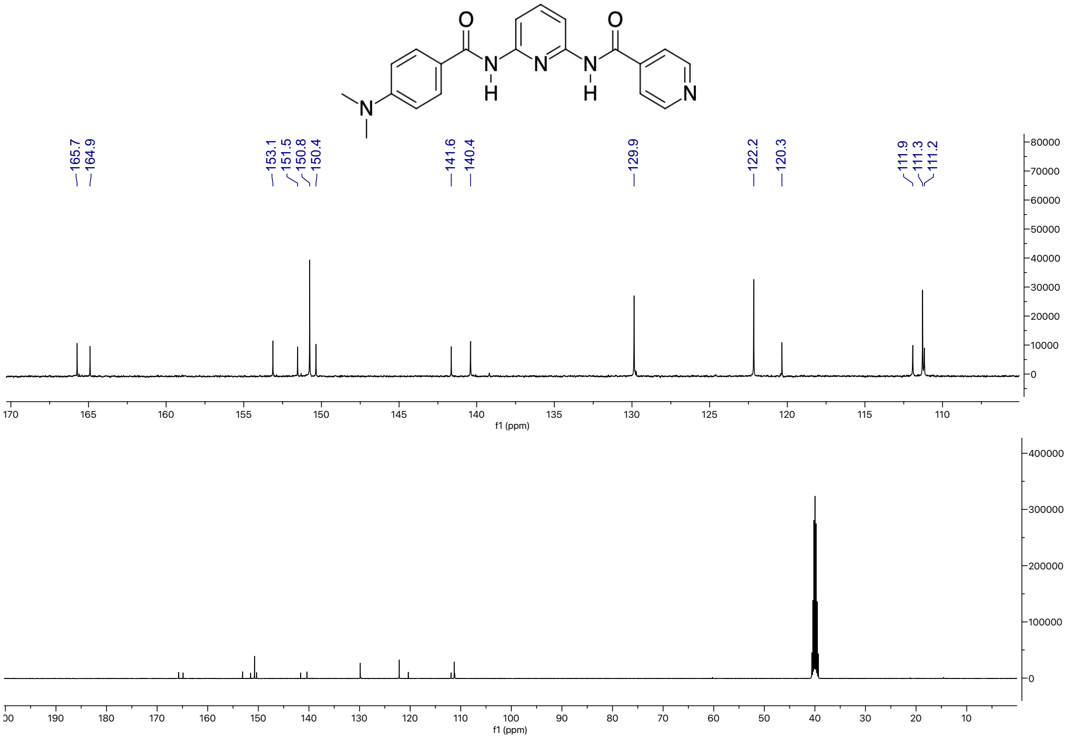

Figure S6: The $^{13}C\{^{1}H\}$ NMR spectrum of bis-amide (101 MHz, DMSO-d_6)

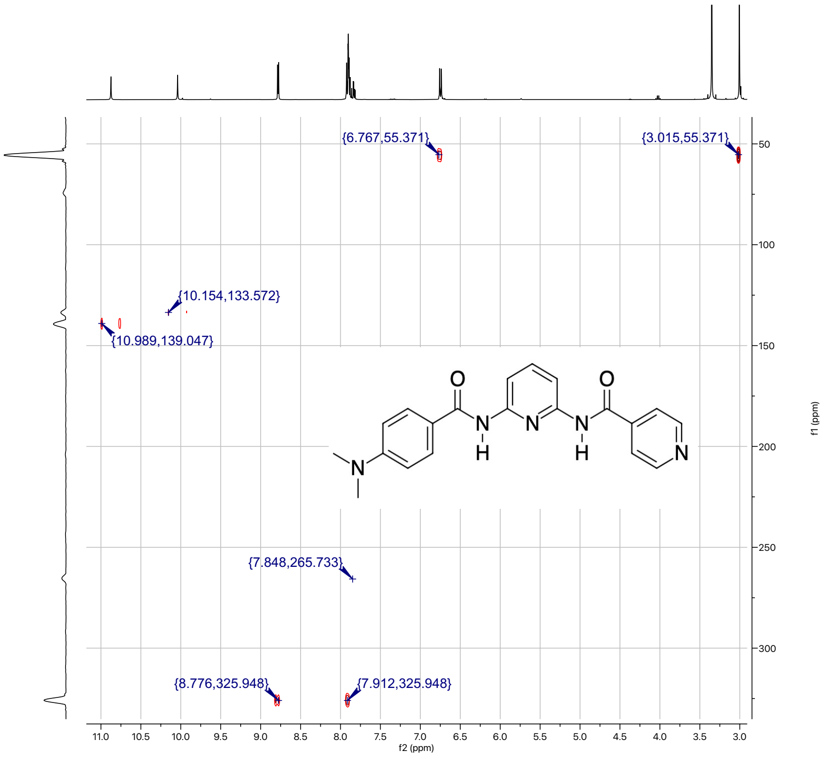

Figure S7: The $^{1}H/^{15}N$ HMBC NMR spectrum of bis-amide (40.5 MHz, DMSO-d_6)

2 Spectroscopic measurements

Table S1: Emission data for **D** in various solvents as a function of temperature

Solvent	Temp.	Wavelength [nm]	Emission intensity[a] [$\times 10^5$]	Intensity ratio[b]
C_6F_6	6 – 60	493 – 483	1.97 – 2.28	1.16
C_6F_5Cl	-14 – 60	489 – 479	3.41 – 3.23	0.95
C_6F_5Br	-26 – 60	500 – 484	2.30 – 2.81	1.22
	-14 – 60	498 – 484	2.48 – 2.81	1.13
C_6F_5I	-26 – 60	534 – 515	0.0948 – 0.3500	3.69
	-14 – 60	536 – 515	0.0977 – 0.3500	3.58

[a]Emission intensity at band maximum

[b]The intensity ratio was calculated dividing the intensity of emission at maximum wavelength at high temperature by the corresponding value at low temperature

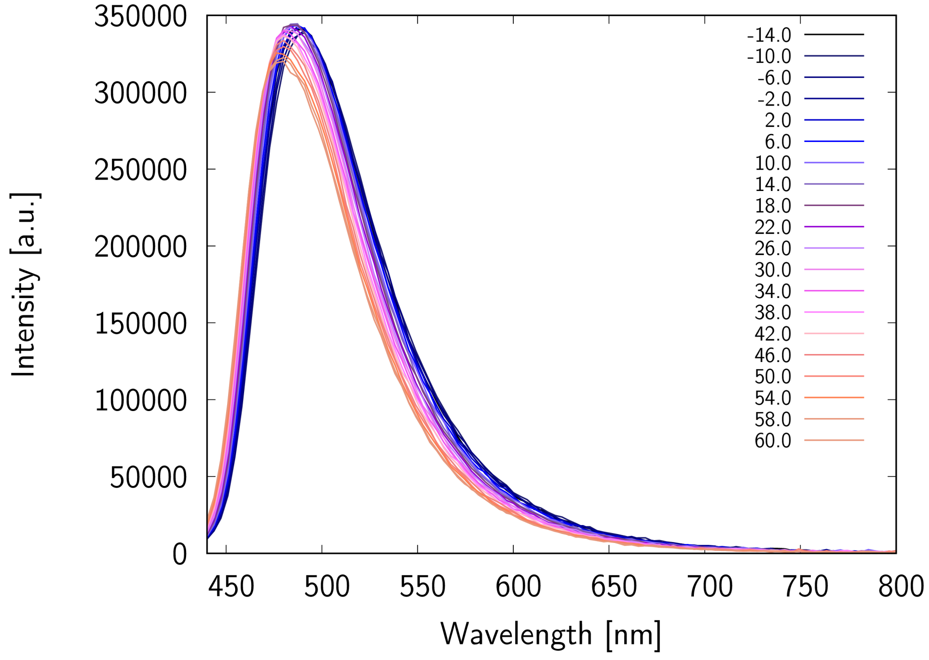

Figure S8: Temperature dependence of fluorescence spectra recorded in C_6F_5Cl

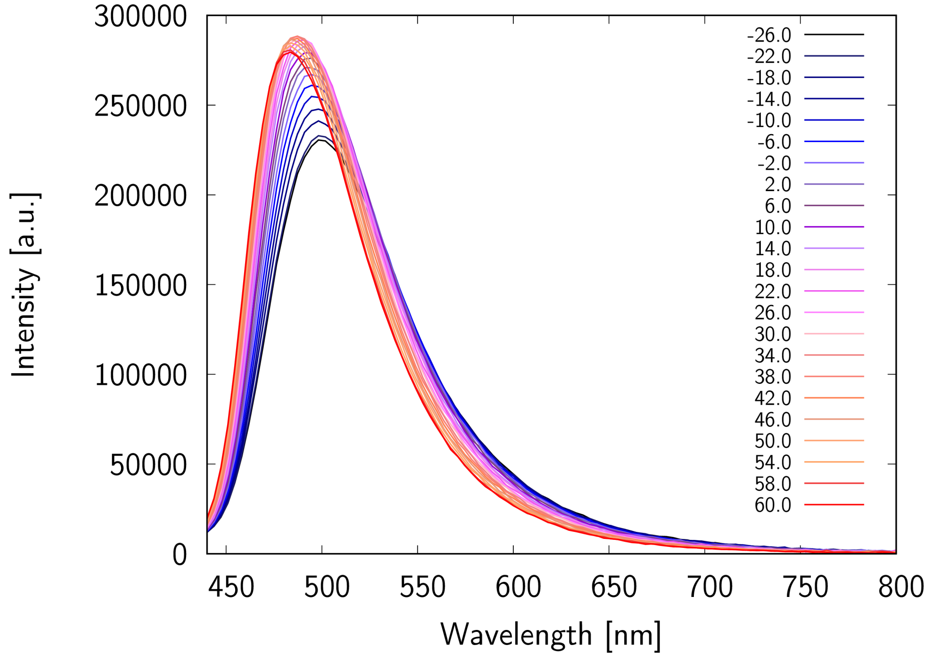

Figure S9: Temperature dependence of fluorescence spectra recorded in C_6F_5Br

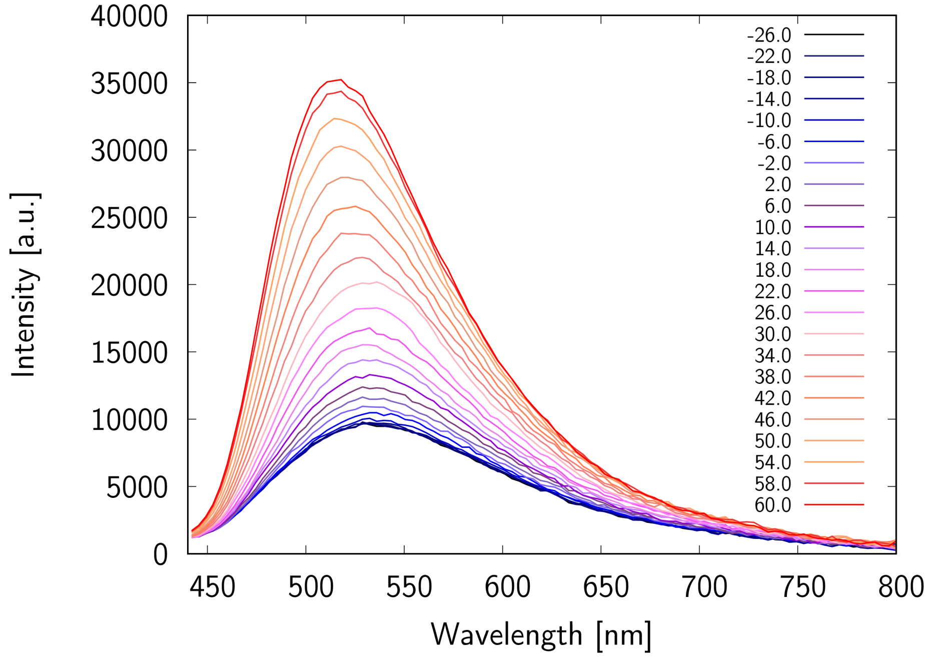

Figure S10: Temperature dependence of fluorescence spectra recorded in C_6F_5I

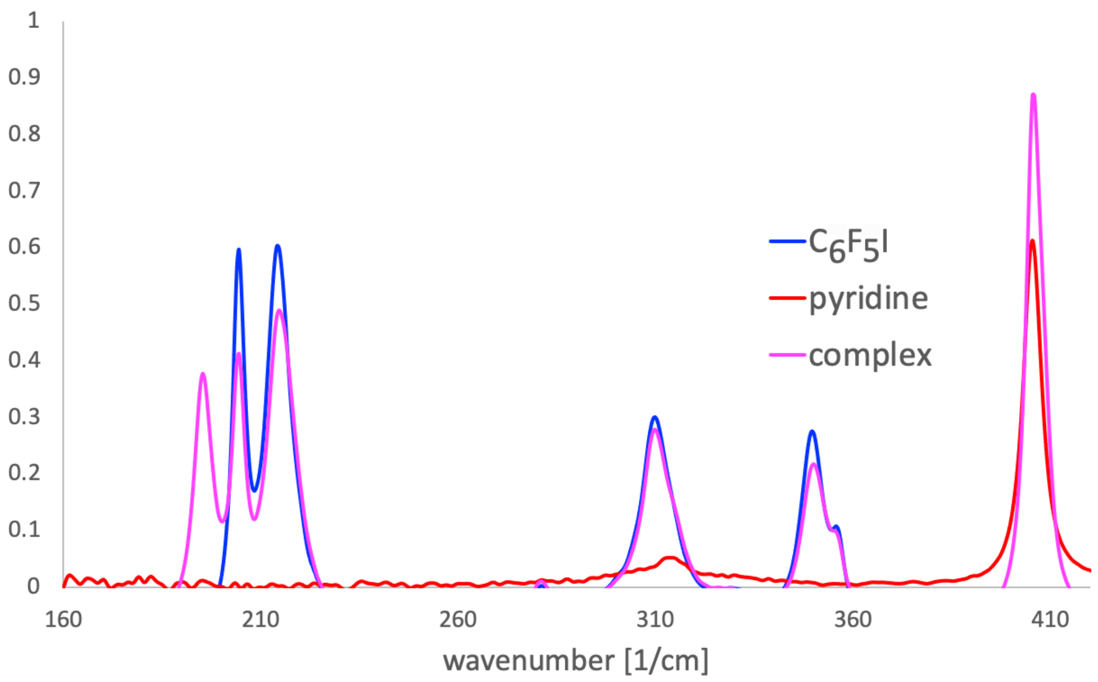

Figure S11: The IR spectrum for pyridine, C_6F_5I and their mixture

The far infrared spectra in the 600-100 cm^{-1} range were collected using a FTIR Bruker VERTEX 70V vacuum spectrometer equipped with an air-cooled DLaTGS detector. The data were collected with 4 cm^{-1} resolution and 64 scans at room temperature. The samples in CCl_4 solution (0.5 M) were placed between poly(ethylene) windows with a path length of 0.5 mm. Given the fact that the solubility of the pyridine-carrying dye (**D**) was low, we measured the IR spectrum for 1:1 mixture of pyridine (model system) and C_6F_5I. The IR spectrum shown in Fig. S11 demonstrates appearance of *new* band at 195 cm^{-1} for the complex which can be assigned to C-I$\cdots$N stretching (note the corresponding value for C-I stretching vibration in C_6F_5I is 204 cm^{-1}). These results are consistent with vibrational-structure calculations at the MN15/aug-cc-pVDZ(PP) level. In the latter case, we find 191 and 201 cm^{-1} for the C_6F_5I:**D** complex and C_6F_5I, respectively. The band assignment for C_6F_5I is also in line with the results obtained by Palmer et al.[S1]

The ^{1}H NMR titration results shown in the main text and in Fig. S12 allowed to study in more detail the interaction of **D** with C_6F_5I in the C_6F_6 solution. This experiment allowed us to find the association constant corresponding to the formation of the complex between the electronic ground state of the dye **D** and C_6F_5I. The association constants based on changes in chemical shifts of protons 1 and 2 are 749 and 767 M^{-1}, respectively. The fit of the obtained data was performed using BindFit[S2] available at WWW.SUPRAMOLECULAR.ORG As seen in the Fig. S15 the CIS values are highest for mentioned nuclei delivering a proof the interaction takes place at the pyridine nitrogen as was assumed during the design of the structure of **D**. The mentioned CIS (*complexation-induced shift*) is the difference between chemical shift of the proton in question at the saturation conditions (i.e. at the end of the titration, when most of the dye molecules are interacting through a XB with C_6F_5I) and the same proton at the beginning of titration (i.e. when the dye is present in the solution as a free molecule). Since the lock of NMR spectrometer needs a deuterated solvent in the solution, the smallest possible volume of C_6D_6 was added. The addition of 60 μL of 99.5% C_6D_6 to 600 μL of C_6F_6 resulted in the appearance of residual C_6H_6 signal at ca. 7.16 ppm. The mentioned amount of C_6D_6 (60μL) was determined is a separate experiment. As a result some signals in **D** are overlapped with C_6H_6. However, for the calculation of the association constant the protons 1 and 2, that are close to the interaction area, safeguard the most accurate results.

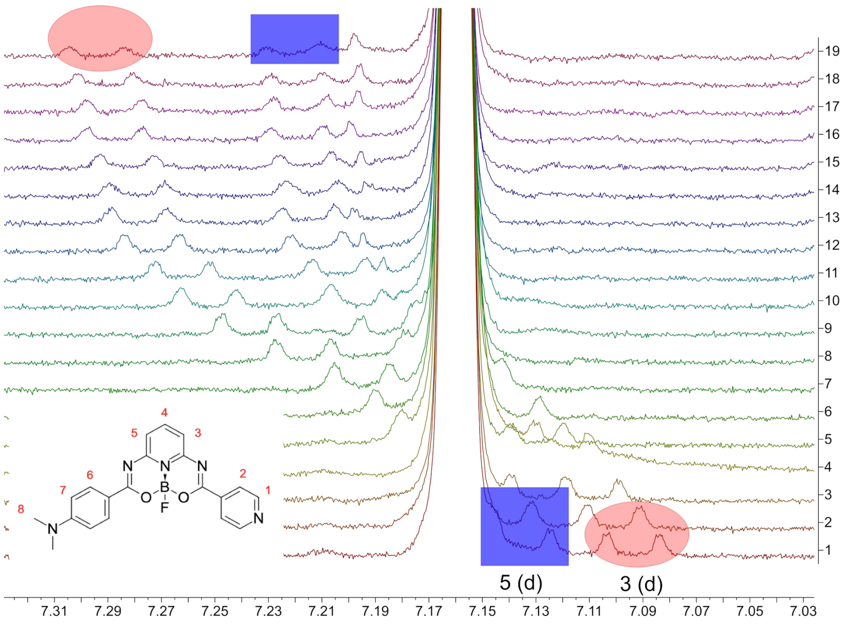

Figure S12: The stacked titration spectra in the region of the residual benzene - visible weak doublets in **D** partially overlapped with benzene

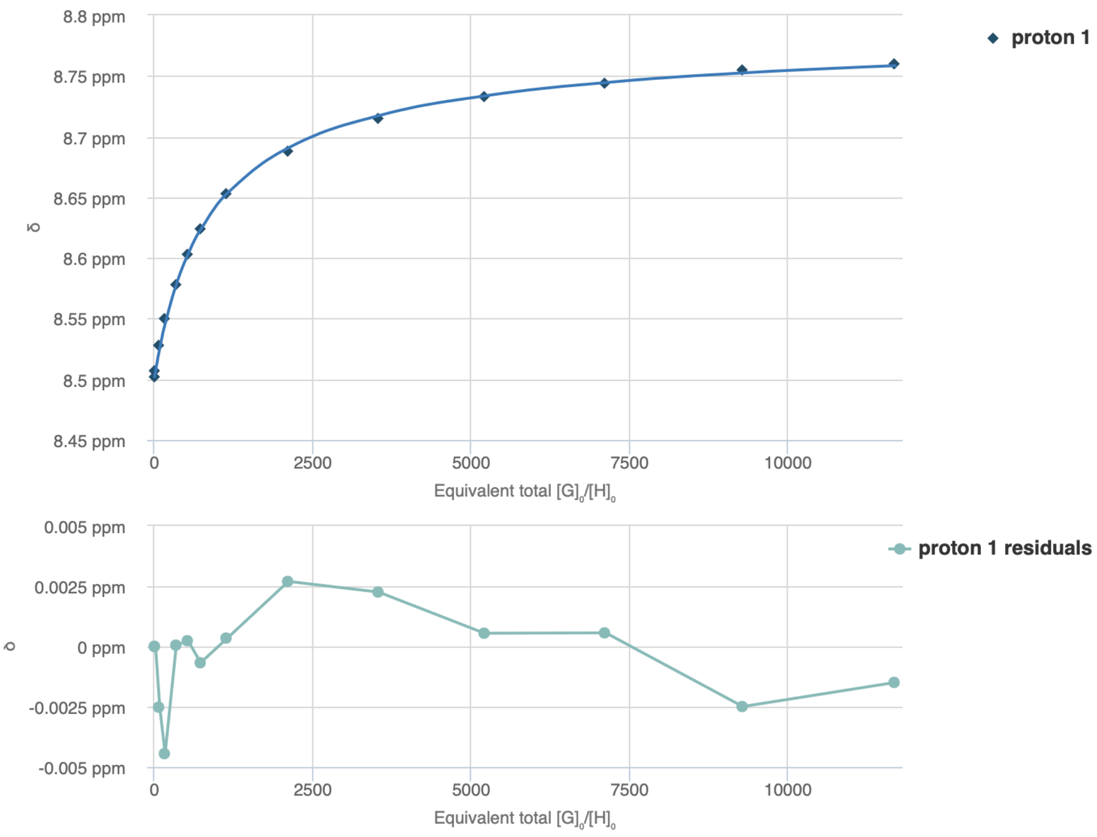

Figure S13: Titration curve for proton no. 1.

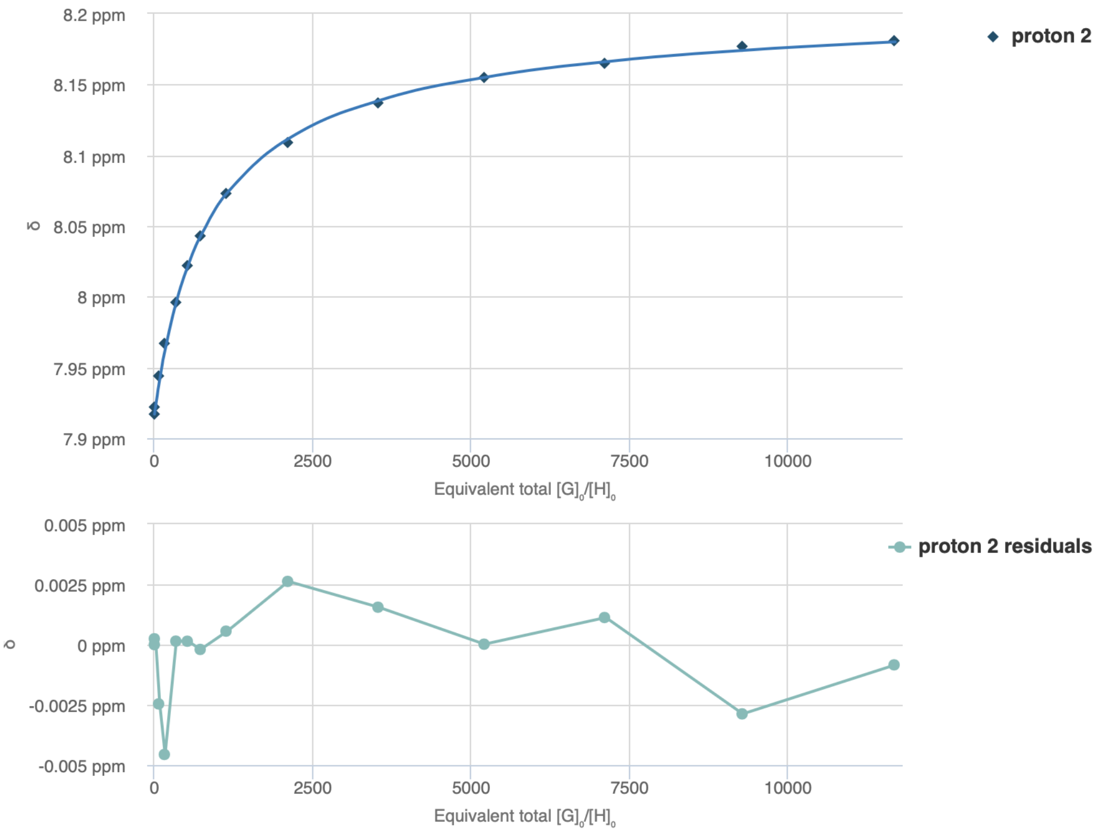

Figure S14: Titration curve for proton no. 2.

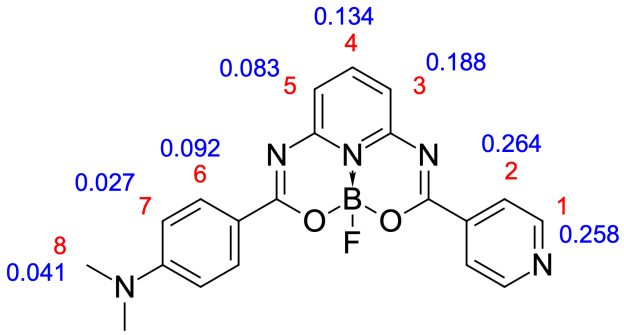

Figure S15: The atom numbering in **D** (in red) and the values of CIS (in blue, see text).

3 Electronic-structure calculations

All results of single-point and vertical absorption/emission calculations can be accessed at:

`https://doi.org/10.19061/iochem-bd-4-39`

on the IOCHEM-BD platform (`www.iochem-bd.org`) to facilitate data exchange and dissemination, according to the FAIR principles of OpenData sharing.

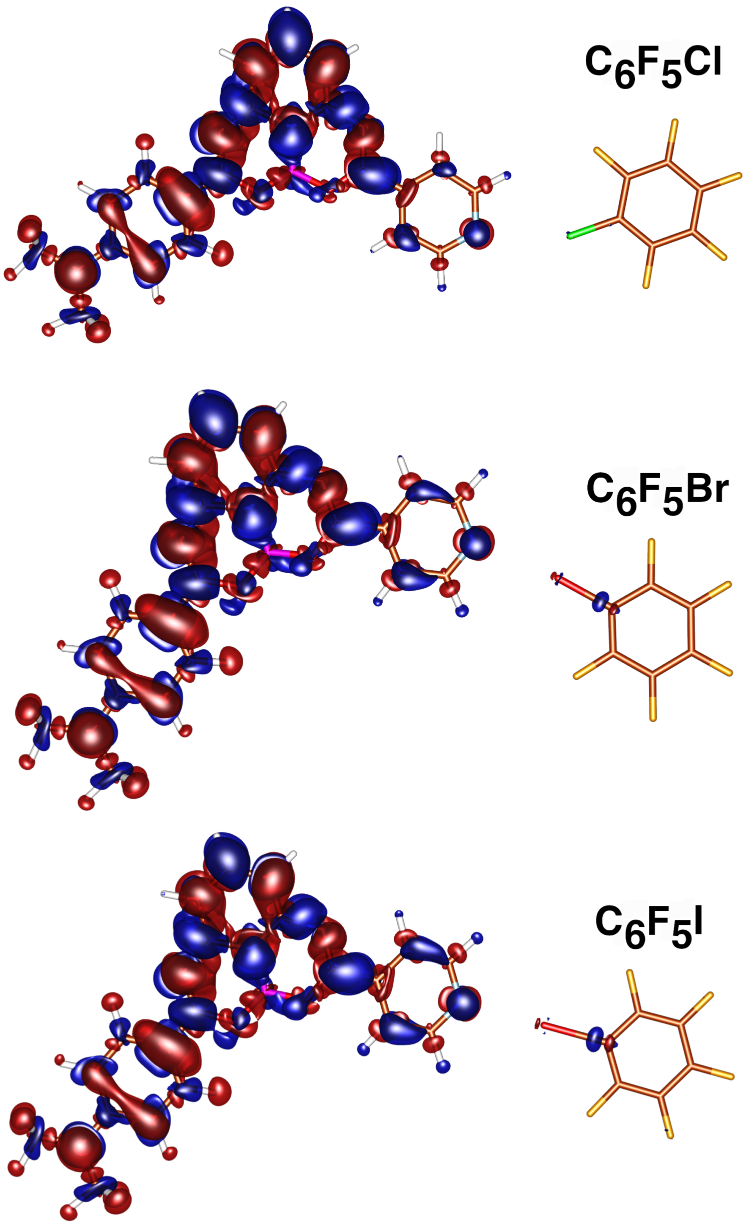

Figure S16: Density difference between the excited and ground states of the vertical absorption process for C_6F_5Cl:**D**, C_6F_5Br:**D** and C_6F_5I:**D** complexes in gas phase at MN15/aug-cc-pVDZ level of theory.

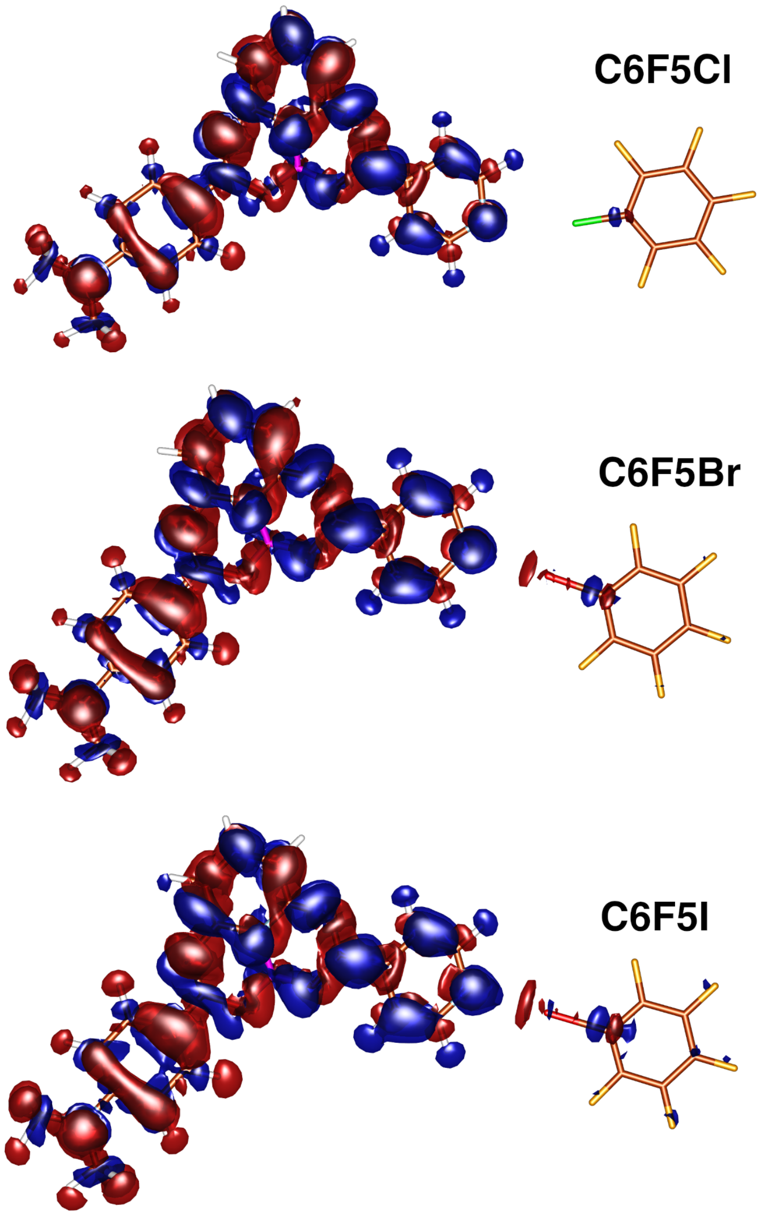

Figure S17: Density difference between the ground and excited states of the vertical emission process for C_6F_5Cl:**D**, C_6F_5Br:**D** and C_6F_5I:**D** complexes in gas phase at MN15/aug-cc-pVDZ level of theory.

Table S2: Intermolecular interaction energy (ΔE, in kcal/mol) computed using SCS-MP2 method and MN15 functional and preparation energy in kcal/mol. The aug-cc-pVDZ(PP) basis set was used during all calculations.

Complex	ΔE (SCS-MP2)	ΔE (MN15)	Preparation energy
C_6F_5Cl:**D**	-2.331	-3.207	0.729
C_6F_5Br:**D**	-4.027	-6.366	1.349
C_6F_5I:**D**	-6.413	-9.227	1.198

Table S3: Computed total energies (a.u.) for ground- and excited state optimized geometries at MN15/aug-cc-pVDZ(PP) level of theory.

Compound	Ground State	Excited State
C_6F_5Cl	-1187.3348287	-
C_6F_5Br	-1144.5041343	-
C_6F_5I	-1022.2093963	-
Dye(**D**)	-1320.1512447	-1320.1433287
C_6F_5Cl:**D**	-2507.4911624	-2507.4824509
C_6F_5Br:**D**	-2464.6653923	-2464.6545243
C_6F_5I:**D**	-2342.3748465	-2342.3620080

Cartesian coordinates of optimized ground state geometries of compounds studied given in Å.

C_6F_5Cl, MN15/aug-cc-pVDZ

Cl	2.831092000	-0.000024000	0.000000000
F	1.054309000	-2.363899000	0.000000000
F	-3.009481000	0.000075000	0.000000000
F	-1.650252000	-2.356594000	0.000000000
F	1.054436000	2.363791000	0.000000000
F	-1.650119000	2.356674000	0.000000000
C	1.115149000	-0.000044000	0.000000000
C	0.407603000	-1.202497000	0.000000000
C	-0.984360000	1.205540000	0.000000000
C	-0.984424000	-1.205495000	0.000000000
C	-1.681398000	0.000040000	0.000000000
C	0.407666000	1.202454000	0.000000000

C_6F_5Br, MN15/aug-cc-pVDZ-PP

Br	2.502541000	0.000000000	-0.000017000
F	0.562401000	-2.366064000	-0.000051000
F	-3.495422000	0.000000000	0.000023000
F	-2.136538000	-2.356470000	-0.000033000
F	0.562401000	2.366063000	0.000044000
F	-2.136538000	2.356471000	0.000062000
C	0.631582000	0.000000000	-0.000004000
C	-0.078206000	-1.199815000	-0.000023000
C	-1.470241000	1.205343000	0.000034000
C	-1.470241000	-1.205343000	-0.000014000
C	-2.167302000	0.000000000	0.000015000
C	-0.078205000	1.199815000	0.000025000

C_6F_5I, MN15/aug-cc-pVDZ-PP

I	-2.297723000	0.000000000	-0.000056000
F	-0.135765000	-2.368297000	0.000003000
F	3.916036000	0.000000000	0.000095000
F	2.558242000	-2.356997000	0.000069000
F	-0.135765000	2.368297000	-0.000010000
F	2.558243000	2.356997000	0.000056000
C	-0.215424000	0.000000000	-0.000006000
C	0.499591000	-1.196856000	0.000015000
C	1.891766000	1.205923000	0.000042000
C	1.891766000	-1.205923000	0.000049000
C	2.587777000	0.000000000	0.000063000
C	0.499591000	1.196856000	0.000009000

dye (**D**), MN15/aug-cc-pVDZ

C	0.198903000	2.473748000	-0.020704000
C	0.458915000	3.834032000	-0.244772000
N	1.256234000	1.624354000	0.100874000
H	-0.386783000	4.511994000	-0.325707000
C	1.776894000	4.260130000	-0.361925000
C	2.552378000	2.008964000	-0.055986000
H	1.986191000	5.316465000	-0.533493000
C	2.837972000	3.354077000	-0.281952000
H	3.876628000	3.652699000	-0.395873000
N	3.556239000	1.066197000	-0.022950000
C	3.239055000	-0.195932000	-0.017549000
O	2.015060000	-0.687649000	0.043577000
B	0.969070000	0.181845000	0.582125000
N	-1.081353000	2.003553000	0.052182000
C	-1.292673000	0.708093000	0.055403000
O	-0.336698000	-0.210295000	0.077228000
C	4.313725000	-1.213481000	-0.118152000
C	5.651724000	-0.828100000	-0.246027000
C	4.009787000	-2.576790000	-0.087573000
C	6.621217000	-1.824925000	-0.337485000
C	5.059308000	-3.491569000	-0.189680000
N	6.342511000	-3.135247000	-0.312595000
H	5.915083000	0.227422000	-0.271773000
H	2.979768000	-2.911386000	0.016163000
H	7.675030000	-1.557350000	-0.436647000
H	4.854435000	-4.563858000	-0.169892000
C	-2.657787000	0.189287000	-0.002876000
C	-2.906318000	-1.191733000	0.048616000
C	-3.752826000	1.063220000	-0.109292000
C	-4.199421000	-1.689411000	-0.005997000
C	-5.050102000	0.581339000	-0.162559000
C	-5.308191000	-0.813107000	-0.113807000
H	-2.065298000	-1.877464000	0.137121000
H	-3.562195000	2.134747000	-0.148324000
H	-4.350707000	-2.765457000	0.039597000
H	-5.871405000	1.289876000	-0.243106000
F	0.995053000	0.163091000	1.974370000
N	-6.590838000	-1.297116000	-0.168399000
C	-7.708201000	-0.380236000	-0.262171000
H	-8.637717000	-0.955645000	-0.291446000
H	-7.749853000	0.298159000	0.604039000
H	-7.653514000	0.230987000	-1.176058000
C	-6.829277000	-2.724474000	-0.105998000
H	-6.342885000	-3.251405000	-0.941120000
H	-6.458829000	-3.156737000	0.836467000
H	-7.905705000	-2.907517000	-0.167219000

C_6F_5Cl:**D**, MN15/aug-cc-pVDZ

C	4.508176000	-2.696750000	-0.047805000
C	4.555590000	-4.073905000	-0.312075000
N	3.292451000	-2.118475000	0.156133000
H	5.528647000	-4.535447000	-0.458810000
C	3.366563000	-4.790189000	-0.383512000
C	2.113065000	-2.788551000	0.045121000
H	3.396488000	-5.861280000	-0.585758000
C	2.131645000	-4.156456000	-0.218898000
H	1.184728000	-4.684116000	-0.295434000
N	0.922624000	-2.104635000	0.161862000
C	0.941410000	-0.804503000	0.201902000
O	2.020532000	-0.043750000	0.223308000
B	3.266558000	-0.661652000	0.678207000
N	5.648176000	-1.945445000	-0.016715000
C	5.556613000	-0.636790000	0.026557000
O	4.417918000	0.035021000	0.128264000
C	-0.343233000	-0.062036000	0.192854000
C	-1.560404000	-0.744749000	0.108160000
C	-0.361240000	1.332956000	0.268801000
C	-2.738000000	0.000034000	0.102933000
C	-1.597006000	1.981111000	0.254067000
N	-2.766935000	1.337984000	0.172956000
H	-1.573752000	-1.831031000	0.048174000
H	0.567454000	1.894828000	0.340694000
H	-3.706624000	-0.501344000	0.038621000
H	-1.645396000	3.070157000	0.311647000
C	6.761443000	0.184071000	-0.075311000
C	6.691480000	1.583160000	0.021400000
C	8.019079000	-0.411463000	-0.270401000
C	7.832239000	2.365707000	-0.074136000
C	9.167451000	0.356581000	-0.366031000
C	9.103728000	1.770966000	-0.270933000
H	5.722821000	2.054942000	0.178086000
H	8.075800000	-1.496423000	-0.345054000
H	7.736736000	3.445926000	0.008316000
H	10.122453000	-0.141845000	-0.516075000
F	3.312114000	-0.686337000	2.069859000
N	10.237237000	2.537827000	-0.365674000
C	11.525954000	1.905780000	-0.559740000
H	12.297070000	2.679746000	-0.607816000
H	11.772340000	1.225456000	0.270001000
H	11.557191000	1.330531000	-1.497856000
C	10.146527000	3.979774000	-0.262606000
H	9.510264000	4.402206000	-1.055444000
H	9.736623000	4.291156000	0.710582000
H	11.148209000	4.406647000	-0.364358000
F	-6.722734000	-1.344158000	-0.162411000
F	-11.348895000	-0.514222000	-0.309444000
F	-9.261611000	-2.261306000	-0.329949000
F	-8.350707000	3.089725000	0.047525000
F	-10.884028000	2.159808000	-0.120214000
C	-7.475924000	0.895280000	-0.053404000
C	-7.730261000	-0.473259000	-0.150682000
C	-9.862584000	1.307265000	-0.130033000
C	-9.033485000	-0.953593000	-0.237307000
C	-10.102091000	-0.060895000	-0.226947000
C	-8.555489000	1.778812000	-0.043921000
Cl	-5.863003000	1.467969000	0.051196000

C_6F_5Br:**D**, MN15/aug-cc-pVDZ-PP

C	5.536974000	-2.633261000	-0.096192000
C	6.080468000	-3.897376000	-0.373391000
N	4.190518000	-2.532887000	0.079426000
H	7.157388000	-3.976990000	-0.497159000
C	5.229416000	-4.989897000	-0.486245000
C	3.332980000	-3.578882000	-0.071465000
H	5.645636000	-5.975030000	-0.698973000
C	3.845508000	-4.843456000	-0.349680000
H	3.152632000	-5.673519000	-0.458437000
N	1.973462000	-3.367954000	0.019538000
C	1.525991000	-2.148318000	0.072903000
O	2.258735000	-1.051810000	0.130477000
B	3.633261000	-1.192118000	0.615647000
N	6.331250000	-1.525376000	-0.025098000
C	5.776645000	-0.336597000	0.029648000
O	4.470624000	-0.119296000	0.106698000
C	0.060258000	-1.914981000	0.037468000
C	-0.828275000	-2.988524000	-0.079537000
C	-0.456825000	-0.619737000	0.120547000
C	-2.193844000	-2.717716000	-0.108234000
C	-1.841683000	-0.456951000	0.079519000
N	-2.697515000	-1.478663000	-0.032135000
H	-0.448638000	-4.005885000	-0.145424000
H	0.207327000	0.236149000	0.217245000
H	-2.917061000	-3.529920000	-0.196955000
H	-2.286266000	0.539052000	0.140607000
C	6.609164000	0.862249000	-0.029973000
C	6.040243000	2.141865000	0.075648000
C	8.001244000	0.759623000	-0.192636000
C	6.826957000	3.282150000	0.019769000
C	8.800278000	1.889147000	-0.247980000
C	8.231773000	3.185302000	-0.143975000
H	4.963330000	2.233064000	0.207492000
H	8.444665000	-0.231703000	-0.274125000
H	6.348881000	4.255054000	0.107670000
H	9.873869000	1.768105000	-0.373614000
F	3.649807000	-1.227726000	2.007381000
N	9.017133000	4.308229000	-0.199225000
C	10.451296000	4.182940000	-0.359957000
H	10.894851000	5.182347000	-0.378412000
H	10.904098000	3.620772000	0.471222000
H	10.709860000	3.674427000	-1.301471000
C	8.413318000	5.620388000	-0.088888000
H	7.688150000	5.800428000	-0.897281000
H	7.894898000	5.747667000	0.873922000
H	9.197831000	6.379094000	-0.158100000
Br	-5.300812000	-0.371602000	-0.050092000
F	-8.161434000	-1.491716000	-0.208681000
F	-10.574160000	2.533772000	-0.041815000
F	-10.517478000	-0.183471000	-0.204299000
F	-5.881860000	2.641830000	0.113328000
F	-8.245317000	3.936136000	0.116524000
C	-6.949189000	0.534762000	-0.047697000
C	-8.154655000	-0.160658000	-0.127904000
C	-8.217972000	2.607138000	0.037261000
C	-9.378769000	0.502313000	-0.126619000
C	-9.409154000	1.891484000	-0.043629000
C	-7.004509000	1.925251000	0.034557000

C_6F_5I:**D**, MN15/aug-cc-pVDZ-PP

C	-5.791008000	2.687146000	-0.121459000
C	-6.200074000	3.997926000	-0.414471000
N	-4.464437000	2.453924000	0.077870000
H	-7.261387000	4.184260000	-0.557030000
C	-5.242416000	4.998824000	-0.518069000
C	-3.504387000	3.408150000	-0.063755000
H	-5.554199000	6.019207000	-0.742815000
C	-3.882191000	4.715438000	-0.356893000
H	-3.108018000	5.471282000	-0.458234000
N	-2.173919000	3.061674000	0.050544000
C	-1.853537000	1.803996000	0.117250000
O	-2.692271000	0.786367000	0.168695000
B	-4.053875000	1.067981000	0.631273000
N	-6.693572000	1.665498000	-0.057990000
C	-6.262509000	0.427224000	0.014206000
O	-4.986026000	0.081077000	0.115379000
C	-0.417669000	1.424111000	0.105293000
C	0.575532000	2.402731000	-0.003587000
C	-0.037177000	0.082990000	0.201221000
C	1.906499000	1.995793000	-0.013052000
C	1.323249000	-0.222057000	0.179497000
N	2.275488000	0.710865000	0.074710000
H	0.300814000	3.452569000	-0.078846000
H	-0.787069000	-0.699414000	0.292282000
H	2.714710000	2.724993000	-0.095483000
H	1.665773000	-1.256334000	0.250149000
C	-7.209250000	-0.682620000	-0.051546000
C	-6.772472000	-2.011496000	0.074871000
C	-8.581238000	-0.443779000	-0.240826000
C	-7.667498000	-3.068379000	0.014089000
C	-9.487489000	-1.488804000	-0.301678000
C	-9.052773000	-2.834075000	-0.176085000
H	-5.712506000	-2.208167000	0.227143000
H	-8.922470000	0.585787000	-0.338665000
H	-7.290194000	-4.083003000	0.118821000
H	-10.541283000	-1.262948000	-0.448308000
F	-4.086257000	1.115875000	2.022096000
N	-9.944793000	-3.873619000	-0.235678000
C	-11.356408000	-3.608183000	-0.424372000
H	-11.896818000	-4.558703000	-0.441713000
H	-11.765362000	-2.994604000	0.393000000
H	-11.546392000	-3.087090000	-1.375269000
C	-9.476646000	-5.238238000	-0.103380000
H	-8.760234000	-5.498200000	-0.897682000
H	-8.989107000	-5.406080000	0.869245000
H	-10.331698000	-5.915739000	-0.178917000
I	4.981801000	-0.026270000	0.010223000
F	7.725327000	1.666951000	-0.243467000
F	11.009104000	-1.685382000	-0.160052000
F	10.316474000	0.942784000	-0.316415000
F	6.482567000	-2.887726000	0.145926000
F	9.079331000	-3.591632000	0.071463000
C	7.014648000	-0.585816000	-0.046051000
C	8.024956000	0.366439000	-0.163654000
C	8.738430000	-2.305681000	-0.004983000
C	9.370530000	0.011107000	-0.203094000
C	9.726311000	-1.332226000	-0.123229000
C	7.400495000	-1.922382000	0.031987000

Cartesian coordinates of optimized excited state geometries (below) of compounds studied given in Å.

dye (**D**), MN15/aug-cc-pVDZ, (S_1 state)

C	0.178517000	2.456077000	-0.029287000
C	0.416145000	3.811130000	-0.243721000
N	1.235891000	1.580154000	0.091741000
H	-0.441601000	4.471913000	-0.345728000
C	1.751792000	4.273353000	-0.353369000
C	2.541040000	1.977245000	-0.088578000
H	1.952026000	5.332185000	-0.509398000
C	2.796632000	3.367066000	-0.289573000
H	3.836924000	3.667261000	-0.399790000
N	3.531987000	1.083944000	-0.093788000
C	3.241413000	-0.220334000	0.010135000
O	2.015816000	-0.706462000	0.207341000
B	0.991957000	0.199547000	0.697881000
N	-1.087632000	1.968872000	0.012820000
C	-1.278885000	0.654598000	0.107384000
O	-0.337824000	-0.255351000	0.247470000
C	4.300515000	-1.197960000	-0.127015000
C	5.632268000	-0.806574000	-0.377856000
C	4.039637000	-2.579441000	-0.020970000
C	6.605691000	-1.789593000	-0.506062000
C	5.095114000	-3.473175000	-0.169492000
N	6.364341000	-3.108397000	-0.409002000
H	5.880826000	0.249083000	-0.466291000
H	3.030410000	-2.934587000	0.177132000
H	7.642466000	-1.504551000	-0.698852000
H	4.910987000	-4.547205000	-0.090155000
C	-2.642163000	0.160804000	0.025314000
C	-2.910684000	-1.222079000	0.133725000
C	-3.727181000	1.047490000	-0.166395000
C	-4.201555000	-1.703534000	0.055193000
C	-5.021988000	0.579922000	-0.243780000
C	-5.297948000	-0.813734000	-0.136296000
H	-2.077377000	-1.905740000	0.284996000
H	-3.515651000	2.111873000	-0.248994000
H	-4.372781000	-2.773507000	0.145232000
H	-5.832455000	1.290252000	-0.388040000
F	1.004495000	0.277068000	2.093570000
N	-6.574893000	-1.283927000	-0.212787000
C	-7.682468000	-0.364812000	-0.405942000
H	-8.614069000	-0.934860000	-0.439461000
H	-7.747077000	0.359292000	0.419095000
H	-7.579489000	0.189447000	-1.350088000
C	-6.840494000	-2.707610000	-0.100949000
H	-6.334627000	-3.270671000	-0.898466000
H	-6.506214000	-3.099893000	0.870330000
H	-7.916713000	-2.874690000	-0.189213000

C_6F_5Cl:**D**, MN15/aug-cc-pVDZ, (S_1 state)

C	-4.685980000	2.690478000	-0.081511000
C	-4.821994000	4.045937000	-0.359425000
N	-3.440523000	2.153834000	0.157211000
H	-5.819069000	4.433487000	-0.554696000
C	-3.661935000	4.861701000	-0.410032000
C	-2.286587000	2.893364000	0.036310000
H	-3.753666000	5.927494000	-0.613764000
C	-2.415708000	4.292484000	-0.227469000
H	-1.495671000	4.870833000	-0.286236000
N	-1.089382000	2.318621000	0.146489000
C	-1.009405000	0.988551000	0.303065000
O	-2.065414000	0.182689000	0.435532000
B	-3.333109000	0.777105000	0.813372000
N	-5.767476000	1.865644000	-0.091889000
C	-5.590084000	0.557044000	0.059103000
O	-4.446899000	-0.047142000	0.300621000
C	0.285553000	0.347621000	0.298814000
C	1.473144000	1.090314000	0.121593000
C	0.413308000	-1.048079000	0.465533000
C	2.688417000	0.418914000	0.120699000
C	1.683153000	-1.612293000	0.445351000
N	2.815704000	-0.910948000	0.276632000
H	1.424914000	2.169163000	-0.010053000
H	-0.469500000	-1.666929000	0.610889000
H	3.619054000	0.976440000	-0.013771000
H	1.800175000	-2.690776000	0.573155000
C	-6.753882000	-0.303305000	-0.078403000
C	-6.633264000	-1.701198000	0.086784000
C	-8.025641000	0.236472000	-0.381096000
C	-7.729680000	-2.528399000	-0.043178000
C	-9.130008000	-0.578076000	-0.511249000
C	-9.013857000	-1.989020000	-0.346676000
H	-5.655574000	-2.116747000	0.323371000
H	-8.112243000	1.313938000	-0.506949000
H	-7.602982000	-3.599592000	0.092938000
H	-10.093537000	-0.130002000	-0.741083000
F	-3.453167000	0.896597000	2.200811000
N	-10.099691000	-2.801039000	-0.474234000
C	-11.403182000	-2.237953000	-0.780969000
H	-12.134078000	-3.047971000	-0.838971000
H	-11.724987000	-1.533640000	-0.000494000
H	-11.390816000	-1.710210000	-1.745403000
C	-9.966720000	-4.237914000	-0.303629000
H	-9.268244000	-4.662267000	-1.038873000
H	-9.607805000	-4.486843000	0.705237000
H	-10.944842000	-4.703041000	-0.447295000
F	7.167120000	1.548668000	-0.353186000
F	11.569177000	-0.100615000	-0.427032000
F	9.823785000	1.982112000	-0.590351000
F	7.984971000	-3.074214000	0.214682000
F	10.638712000	-2.626652000	-0.024133000
C	7.507340000	-0.774209000	-0.062900000
C	8.001263000	0.514642000	-0.269797000
C	9.784360000	-1.608535000	-0.103674000
C	9.368119000	0.746695000	-0.393029000
C	10.261935000	-0.317304000	-0.309745000
C	8.415578000	-1.830634000	0.018330000
Cl	5.821273000	-1.048994000	0.087796000

C_6F_5Br:**D**, MN15/aug-cc-pVDZ-PP, (S_1 state)

C	5.402574000	-2.685725000	-0.117548000
C	5.812155000	-3.979846000	-0.389965000
N	4.073243000	-2.400231000	0.078439000
H	6.870508000	-4.166932000	-0.553023000
C	4.829448000	-5.005132000	-0.474643000
C	3.085246000	-3.345345000	-0.071656000
H	5.132954000	-6.032249000	-0.673192000
C	3.496241000	-4.694356000	-0.328578000
H	2.710235000	-5.442860000	-0.407809000
N	1.798344000	-3.018362000	0.009096000
C	1.450549000	-1.728956000	0.152805000
O	2.332709000	-0.728769000	0.281185000
B	3.672904000	-1.060436000	0.705569000
N	6.307573000	-1.658035000	-0.090435000
C	5.871634000	-0.417248000	0.037281000
O	4.626006000	-0.039304000	0.212105000
C	0.061321000	-1.355530000	0.131747000
C	-0.958862000	-2.319522000	-0.048384000
C	-0.341775000	-0.008133000	0.283096000
C	-2.280198000	-1.902308000	-0.067800000
C	-1.695110000	0.295800000	0.245392000
N	-2.663634000	-0.619540000	0.073380000
H	-0.698556000	-3.368811000	-0.168508000
H	0.400049000	0.773609000	0.429647000
H	-3.084309000	-2.628957000	-0.204374000
H	-2.029366000	1.329594000	0.360420000
C	6.857282000	0.655430000	-0.048956000
C	6.458898000	2.002257000	0.102254000
C	8.222288000	0.369078000	-0.285259000
C	7.379532000	3.025359000	0.022934000
C	9.154012000	1.380152000	-0.364551000
C	8.758437000	2.742842000	-0.213178000
H	5.408416000	2.217488000	0.287353000
H	8.517234000	-0.671996000	-0.400339000
H	7.043272000	4.051683000	0.146549000
H	10.196096000	1.127966000	-0.543851000
F	3.781215000	-1.139001000	2.097311000
N	9.670591000	3.747366000	-0.290103000
C	11.073834000	3.449280000	-0.527667000
H	11.635149000	4.385867000	-0.552812000
H	11.483755000	2.817311000	0.272454000
H	11.210519000	2.933865000	-1.488560000
C	9.256774000	5.133108000	-0.134438000
H	8.528377000	5.416646000	-0.906690000
H	8.806999000	5.302320000	0.853737000
H	10.133862000	5.776700000	-0.231471000
Br	-5.286141000	0.084369000	-0.000563000
F	-7.798556000	-1.672523000	-0.339918000
F	-11.111423000	1.651214000	-0.174315000
F	-10.395539000	-0.965904000	-0.416780000
F	-6.594062000	2.868973000	0.226307000
F	-9.194988000	3.560287000	0.147543000
C	-7.109305000	0.574661000	-0.053925000
C	-8.111473000	-0.380548000	-0.217385000
C	-8.845501000	2.279364000	0.028973000
C	-9.458406000	-0.031300000	-0.259222000
C	-9.825702000	1.305283000	-0.135417000
C	-7.505080000	1.905804000	0.067765000

C_6F_5I:**D**, MN15/aug-cc-pVDZ-PP, (S_1 state)

C	5.747480000	-2.677743000	-0.124938000
C	6.173971000	-3.962055000	-0.396270000
N	4.417539000	-2.404722000	0.066001000
H	7.234163000	-4.140064000	-0.555554000
C	5.198382000	-4.996517000	-0.482275000
C	3.437133000	-3.357435000	-0.080976000
H	5.512796000	-6.020805000	-0.679886000
C	3.864534000	-4.703005000	-0.336888000
H	3.088028000	-5.461490000	-0.413758000
N	2.147403000	-3.045371000	0.003685000
C	1.782163000	-1.759678000	0.139668000
O	2.658197000	-0.747294000	0.246664000
B	3.997159000	-1.059256000	0.675738000
N	6.645127000	-1.632802000	-0.092284000
C	6.192617000	-0.404180000	0.020223000
O	4.943069000	-0.032666000	0.172318000
C	0.393391000	-1.401816000	0.129508000
C	-0.621184000	-2.378409000	-0.033411000
C	-0.024276000	-0.055315000	0.273087000
C	-1.945145000	-1.977305000	-0.046010000
C	-1.378226000	0.235968000	0.244214000
N	-2.337684000	-0.694281000	0.087532000
H	-0.351583000	-3.425993000	-0.146626000
H	0.710127000	0.735456000	0.406573000
H	-2.745382000	-2.710290000	-0.169753000
H	-1.725921000	1.266004000	0.352682000
C	7.170941000	0.683590000	-0.056851000
C	6.752221000	2.025468000	0.080507000
C	8.542484000	0.409328000	-0.269551000
C	7.661312000	3.058668000	0.010490000
C	9.463482000	1.429622000	-0.339387000
C	9.048136000	2.789411000	-0.201697000
H	5.696093000	2.226703000	0.247164000
H	8.848030000	-0.629762000	-0.373696000
H	7.312001000	4.081833000	0.122474000
H	10.511262000	1.189500000	-0.500460000
F	4.112798000	-1.121039000	2.067906000
N	9.948277000	3.802639000	-0.269078000
C	11.360128000	3.521521000	-0.482035000
H	11.909335000	4.464999000	-0.502666000
H	11.762582000	2.898870000	0.328524000
H	11.517151000	3.003209000	-1.437756000
C	9.517469000	5.185999000	-0.128030000
H	8.799428000	5.455600000	-0.914225000
H	9.049848000	5.353672000	0.851598000
H	10.389268000	5.837756000	-0.214035000
I	-4.924895000	0.011028000	0.013278000
F	-7.693264000	-1.663069000	-0.313400000
F	-10.970475000	1.694316000	-0.181138000
F	-10.280237000	-0.931420000	-0.396158000
F	-6.445296000	2.879323000	0.204548000
F	-9.038048000	3.589805000	0.119438000
C	-6.972845000	0.581131000	-0.051037000
C	-7.986980000	-0.361667000	-0.203516000
C	-8.697903000	2.303618000	0.014355000
C	-9.331976000	-0.004293000	-0.248869000
C	-9.686866000	1.336771000	-0.139136000
C	-7.361717000	1.914458000	0.056053000

References

(S1) Palmer, M. H.; Biczysko, M.; Peterson, K. A.; Stapleton, C. S.; Wells, S. P. Structural and Vibrational Properties of Iodopentafluorobenzene: A Combined Raman and Infrared Spectral and Theoretical Study. *J. Phys. Chem. A* **2017**, *121*, 7917–7924.

(S2) Brynn Hibbert, D.; Thordarson, P. The death of the Job plot, transparency, open science and online tools, uncertainty estimation methods and other developments in supramolecular chemistry data analysis. *Chem. Commun.* **2016**, *52*, 12792–12805.
